# Gene deletion as a possible strategy adopted by New World *Leishmania infantum* to maximize geographic dispersion

**DOI:** 10.1371/journal.ppat.1012938

**Published:** 2025-03-20

**Authors:** Monique Florêncio, Marne Coimbra Batalha Chagas, Anderson Guimarães-Costa, Jullyanna Oliveira, Ingrid Waclawiak, Thamara K. F. Oliveira, Elvira Maria Saraiva, Anita Leocadio Freitas-Mesquita, José Roberto Meyer-Fernandes, Laura Aragão-Farias, Camilly Enes Trindade, Patricia Cuervo, Renata Azevedo do Nascimento, Otacilio C. Moreira, Flávia Lima Ribeiro-Gomes, Yara M. Traub-Csekö, Erich Loza Telleria, Slavica Vaselek, Tereza Leštinová, Petr Volf, Gerald F. Späth, Elisa Cupolillo, Mariana Côrtes Boité

**Affiliations:** 1 Laboratório de Pesquisa em Leishmanioses, Instituto Oswaldo Cruz, FIOCRUZ, Rio de Janeiro, Brazil; 2 Laboratório de Imunologia das Leishmanioses, Instituto de Microbiologia Paulo de Góes, Centro de Ciências da Saúde, Universidade Federal do Rio de Janeiro, Rio de Janeiro, Brazil; 3 Instituto de Bioquímica Médica Leopoldo de Meis (IBqM), Universidade Federal do Rio de Janeiro, Rio de Janeiro, Brazil; 4 Laboratory of Molecular Virology and Parasitology, Instituto Oswaldo Cruz, FIOCRUZ, Rio de Janeiro, Brazil; 5 Laboratório de Pesquisa em Malaria, Instituto Oswaldo Cruz, FIOCRUZ, Rio de Janeiro, Brazil; 6 Laboratório de Biologia Molecular de Parasitas e Vetores, Instituto Oswaldo Cruz, Rio de Janeiro, Brazil; 7 Department of Parasitology, Faculty of Science, Charles University, Prague, Czech Republic; 8 Institut Pasteur, Univerisité Paris Cité, INSERM U1201, Unité de Parasitologie Moléculaire et Signalisation, Paris, France; 9 Instituto Nacional de Ciência e tecnologia EpiAMO (INCT). Porto Velho, Brazil; UT Southwestern: The University of Texas Southwestern Medical Center, UNITED STATES OF AMERICA

## Abstract

**Background:**

The present study investigates implications of a sub-chromosomal deletion in *Leishmania infantum* strains, the causative agent of American Visceral Leishmaniasis (AVL). Primarily found in New World strains, the deletion leads to the absence of the ecto-3’-nucleotidase/nuclease enzyme, impacting parasite virulence, pathogenicity, and drug susceptibility. The factors favoring prevalence and the widespread geographic distribution of these deleted mutant parasites (DEL) in the NW (NW) are discussed under the generated data.

**Methods:**

We conducted phenotypic assessments of the sub-chromosomal deletion through *in vitro* assays with axenic parasites and experimental infections in both *in vitro* and *in vivo* models of vertebrate and invertebrate hosts using geographically diverse mutant field isolates.

**Results:**

Despite reduced pathogenicity, the DEL strains efficiently infect vertebrate hosts and exhibit relevant differences, including enhanced metacyclogenesis and colonization rates in sand flies, potentially facilitating transmission. This combination may represent a more effective way to maintain and disperse the transmission cycle of DEL strains.

**Conclusions:**

Phenotypic assessments reveal altered parasite fitness, with potential enhanced transmissibility at the population level. Reduced susceptibility of DEL strains to miltefosine, a key drug in VL treatment, further complicates control efforts. The study underscores the importance of typing parasite genomes for surveillance and control, advocating for the sub-chromosomal deletion as a molecular marker in AVL management.

## Author summary

Leishmaniasis is a serious disease affecting people in the Americas and other continents caused by a parasite called *Leishmania infantum*. This study explores how a specific genetic change in the parasite, known as a sub-chromosomal deletion, affects its behavior. This deletion leads to the loss of an enzyme called ecto-3’-nucleotidase/nuclease, which plays a role in the parasite’s biology and ability to cause disease and respond to treatment. The study examined how this genetic change influences the parasite’s ability to infect cells and the sand flies that spread the parasite. They found that, although these mutant parasites (called DEL strains) are less harmful in some ways, they are better at completing their life cycle in sand flies and can thus spread more efficiently. Additionally, these DEL strains are less susceptible to miltefosine, a key drug used to treat the disease, making it harder to control. The findings suggest that the genetic change helps the parasite spread and survive, despite reducing its ability to infect cells. The study emphasizes the importance of identifying these genetic changes in studies that perform disease tracking and evaluate treatment strategies, suggesting that the deletion in American parasites could serve as a marker for better managing this challenging disease.

## Introduction

*L. infantum* is one of the causative agents of Visceral Leishmaniasis (VL) across Asia, Africa and Europe, and is exclusively responsible for American Visceral Leishmaniasis (AVL), affecting both humans and dogs, the primary urban reservoir. The course of this parasite’s evolution, adaptation and geographic dispersion is constantly under the effect of multivariable interactions in the transmission cycle, which include vertebrate hosts and various sand fly species as vectors. Within this process, the parasite’s intrinsic genome instability has been predicted as a driver in *Leishmania* fitness gain in response to environmental changes [1] or drug pressure [2]. The loss of genes, for instance, is a molecular strategy eventually compensated by adjustments at the genomic, transcriptomic, and post-transcriptomic levels, leading to phenotypic variances [3] that may favor parasite survival and transmission. In this context, our previous study has documented the prevalent distribution of New World (NW) *L. infantum* strains harboring a 12 Kb sub-chromosomal deletion (DEL mutant strains) [4]; noteworthy, this genomic trait is primarily confined to parasite populations in the Americas. The deletion spans across the four copies of tetrasomic chromosome 31 (chr31), presenting stable chromosomal amplification within the mosaic aneuploidy of *Leishmania* parasites [5]. Thus, the loss of all four alleles further supports strong, environmental selection, suggesting that the deletion provides a fitness advantage for *L. infantum* in Brazil.

Four open reading frames (ORF) are covered by the deleted site: LinJ.31.2370 (ecto-3′-nucleotidase/nuclease), LinJ.31.2380 (ecto-3’-nucleotidase precursor), LinJ.31.2390 (helicase-like protein) and LinJ.31.2400 (3,2-trans-enoyl-CoA isomerase). Although the combined effect of the loss of the four genes cannot be underscored, genomic and previous data suggest the specific absence of the two copies of 3’NU/NT could lead to the most prominent phenotypic variations. This claim is based on the fact that only one extra copy is annotated at chromosome 12 and because a relevant associated phenotypic change is reported [4], i.e., the absence of the ecto-3’-nucleotidase/nuclease (3’NU/NT) activity [4] among the mutants. The 3’NU/NT is unique to trypanosomatids [6], and, as an ecto-enzyme, acts upon substrates present extracellularly. Its role encompasses the hydrolysis of extracellular 3′ nucleotides and nucleic acids, generating nucleosides that can be translocated across plasma membranes via nucleoside transporters (NT) [6]. The 3’NU/NT enzyme is involved in the purine salvage pathway of *Leishmania* [ [ [7,8] (trypanosomatids are unable to synthetize purine de novo [7]), thereby influencing nutrition [9]and parasite replication. Moreover, it is implicated as a virulence factor affecting the parasite’s ability to infect macrophages [10–12]and survive neutrophil extracellular traps (NETs) [13,14]. Recent evidence has linked 3’NU/NT to susceptibility to miltefosine (MIL) [15], a critical drug in leishmaniasis treatment, and clinical trials have shown a correlation between reduced MIL efficacy in AVL treatment and infection by DEL strains [16].

The prevalence and geographical spread of DEL strains in Brazil thus underscore a paradoxical hypothesis suggesting that the loss of these genes confers fitness advantages, facilitating the spread of the mutant population. This puzzling association raises fundamental questions, especially regarding the absence of the 3’NU/NT activity, on how the loss of an important virulence and biological function contributes to the high prevalence of the mutant DEL strains. We postulate that the genetic and biological differences revealed so far between DEL and non-deleted strains (NonDEL) alter transmissibility of these parasite genotypes by affecting colonization parameters in the vector, for instance, and/or resulting in variable infection outcomes in vertebrate hosts. To explore this hypothesis, we conducted phenotypic assessments of the sub-chromosomal deletion through *in vitro* assays with axenic parasites and experimental infections in both *in vitro* and *in vivo* models of vertebrate and invertebrate hosts using geographically diverse NonDEL and DEL field isolates.

Our findings reveal that DEL mutant parasites exhibit enhanced metacyclogenesis *in vitro* but diminished capacity to survive neutrophil NETs and infect macrophages. Although the uptake by macrophages of DEL parasites is reduced, the intracellular development and proliferation was not affected. *In vivo* analyses of selected DEL strains demonstrate often reduced recruitment of monocytes/neutrophils in a murine ear infection model. Moreover, colonization parameters in different vector species exhibit notable variances, underscoring the significant influence of sand flies on parasite distribution and indicating a fitness advantage for the intravectorial life stage of DEL strains. Collectively, these data present a spectrum of phenotypes that, while individually possibly diminishing DEL parasite fitness, collectively contribute to enhanced transmissibility, as metacyclic forms are the infective stage transmitted by sand flies, and reduced pathogenicity in the vertebrate host may facilitate transmission [17]. Importantly, the elevated colonization rate observed for DEL strains on key vector species enhances the likelihood of prevalence among these mutants in the Americas.

Further epidemiological implications related to the co-circulation of these *L. infantum* populations for AVL treatment and control is the reduced susceptibility to MIL observed for DEL parasites [16,18]. MIL is currently approved to be utilized in the treatment of canine visceral leishmaniasis (CVL), prompting concerns regarding surveillance and control [19,20], since parasite population is reported as the same among human and dogs. Combined to the altered pathogenicity and infectivity described herein, the data emphasize the importance of detecting asymptomatic individuals and typing the infecting parasite’s genome as DEL or NonDEL, particularly for canine leishmaniasis. Consequently, we advocate for the sub-chromosomal deletion as a key molecular marker for VL surveillance in the American continent.

## Results

### Deletion-carrying strains present relevant biological differences

Initially, a set of 23 culture-adapted *L. infantum* strains, comprising nine deletion-carrying (DEL), three heterozygous (HTZ), and eleven non-deleted (NonDEL) strains, were evaluated. These strains were isolated from humans and dogs, with twenty-two originating from various regions of Brazil and one from Portugal. Subsequently, subsets of strains were chosen for different assays, as outlined in the S1 Fig. Genotypes were previously identified via whole genome sequencing (WGS) [4] and further confirmed by PCR.

**Fig 1:**
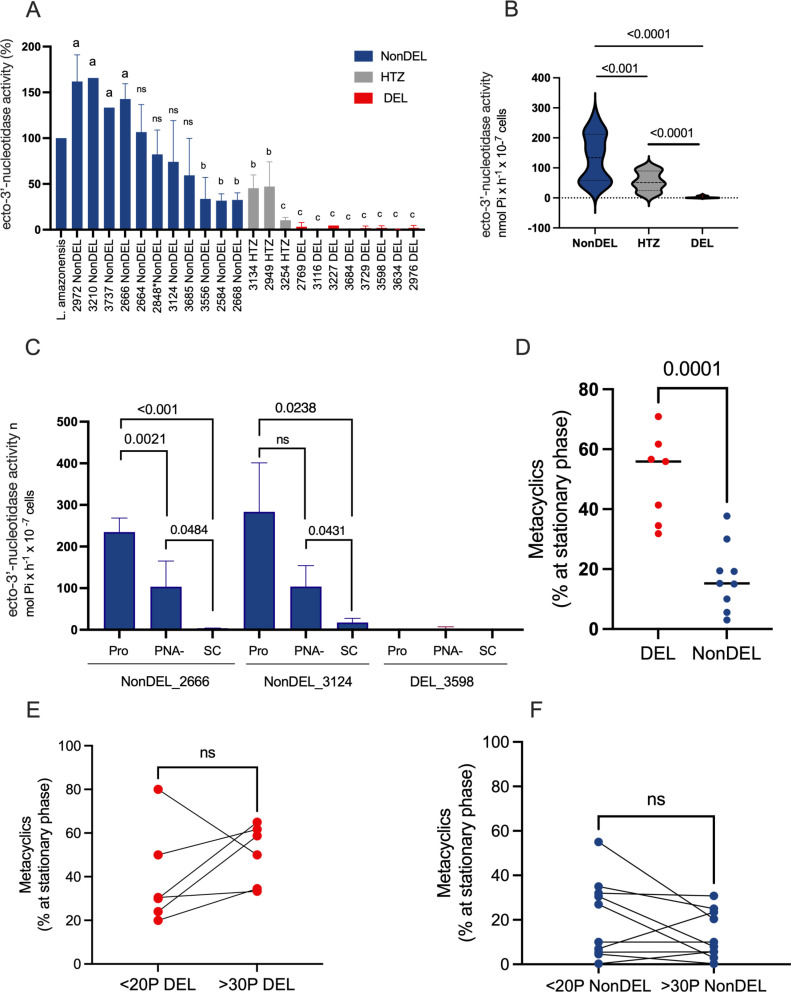
In vitro phenotypic characterization of DEL, NonDEL and HTZ strains. A) Normalized 3’NT activity for NonDEL, HTZ and DEL strains. The 3’NT activity was measured for DEL (n = 08), HTZ (n = 03) and NonDEL (n = 11) parasites harvested at stationary phase. The original values, expressed as nmol Pi x h^-1^ x 10 ^-7^ cells, were normalized by the values obtained with the L. amazonensis assays. Compared to the L. amazonensis control, four NonDEL samples presented higher activity (a); three NonDEL and two HTZ strains presented lower activity (b); all DEL and one HTZ samples presented the lowest enzyme activity (c). Unpaired t-test. B) 3’NT activity average expressed as nmol Pi x h^-1^ x 10 ^-7^ for each genotype group composed by the strains individually presented in graph A. Kruskal-Wallis test followed by Mann-Whitney U test. C) 3’NT activity expressed as nmol Pi x h^-1^ x 10 ^-7^ cells in cultures from: stationary phase promastigotes = Pro; metacyclic enriched fraction of stationary phase culture = PNA−; SC = stressed culture parasites “axenic amastigotes”. D) Percentage of metacyclic at stationary phase. Dots represent the average value of two independent experiments for each strain (DEL = 07 and NonDEl = 09). Unpaired t-Test. E-F) Percentage of metacyclic at stationary phase of less-than-20 passages (<20P) culture and culture-adapted parasites - more than 30 passages (>30P). Percentage of metacyclic was determined 72 hours (late stationary phase) after an initial inoculum of 10^6^ parasites/ml. Metacyclic enrichment was obtained by Peanut agglutinin (PNA); percentage of cells from the PNA− fraction was determined in relation to the total cell count. Red = DEL; Blue = NonDEL; Grey = HTZ. Unpaired t test. E-F) Paired t-test for DEL and NonDEL groups of parasites in <20P and >30P conditions. ns = not-significant. Moderate to strong negative correlation (Pearson r = 0.64)was detected for 3’NT activity (A) and metacyclic enrichment (D) (n =14 strains; S1 Data).

The diminished or absent ecto-3’-nucleotidase activity in deletion-carrying strains (DEL plus HTZ) was confirmed across the entire sample panel, while NonDEL strains exhibited variable activity compared to a *L. amazonensis* reference strain. HTZ strains displayed intermediate activity (Fig 1B), reflecting their intermediate copy number of 3’NU/NT as previously confirmed by qPCR and read depth within the deleted site, as confirmed by WGS [4].

Three strains were selected for assays using promastigotes (Pro), PNA-enriched metacyclic, and pH/temperature stress-exposed parasites (SC) to compare enzyme activity. NonDEL promastigote cultures showed the highest 3’NT activity (Pro; 237.7 and 283.1 nmol Pi x h-1 x 10^−7^ cells), followed by metacyclic forms (103.4 and 103.8 nmol Pi x h-1 x 10^−7^cells). These findings support previous reports indicating significant 3’NU/NT activity among metacyclic forms, suggesting the enzyme’s potential relevance during the intravectorial phase or early infection stages in the vertebrate host. However, enzyme activity in tissue-derived amastigotes has not been measured to date. Notably, cultures exposed to pH/temperature stress (SC) displayed reduced or absent activity (3.3 and 17.4 nmol Pi x h-1 x 10^−7^ cells; Fig 1C). As expected, the DEL sample exhibited no activity across any lifecycle stage (Pro = 0 and PNA− = 3.3 nmol Pi x h-1 x 10^−7^ cells) or in pH/temperature stress exposed cultures.

Low pH and nutritional stress, including the absence of purines [21,22], trigger metacyclogenesis in trypanosomatides [22,23]. Trypanosomes and *Leishmania* lack a de novo purine biosynthetic pathway [7], therefore, these parasites rely on alternative purine salvage routes involving enzymes such as the 3NU/NT. Reports [6,12] suggest that the lack of 3’NT activity affects the parasite nutritional state and replication, thereby triggering metacyclogenesis. That information led us to test whether the parasites with distinct 3’NT activity levels differed in metacyclogenesis and cell density in vitro. Assays with inoculum-controlled cultures (N=16 strains) revealed higher percentages of metacyclic forms in DEL strains (median=56%) compared to NonDEL strains (median = 15.3%), consistent across culture passages (fewer than 20 passages = <20P and more than 30 passages in culture =>30P, S2 Fig). Further paired analysis confirmed no notable variation in metacyclogenesis between <20P and >30P cultures (Fig 1E-F). Thus, despite the acknowledged impact of culture maintenance on *Leishmania* biology, the contrast in metacyclogenesis between DEL and NonDEL parasites remains consistent. Moreover, a moderate to strong negative correlation (Pearson r = −0.64) was obtained for the 3’NT activity and the percentage of metacyclic forms in vitro (n = 14 strains; S1 Data), supporting the association between these phenotypes.

### Higher abundance of transcripts of nucleoside transporter 1 and META2 among DEL strains

**Fig 2:**
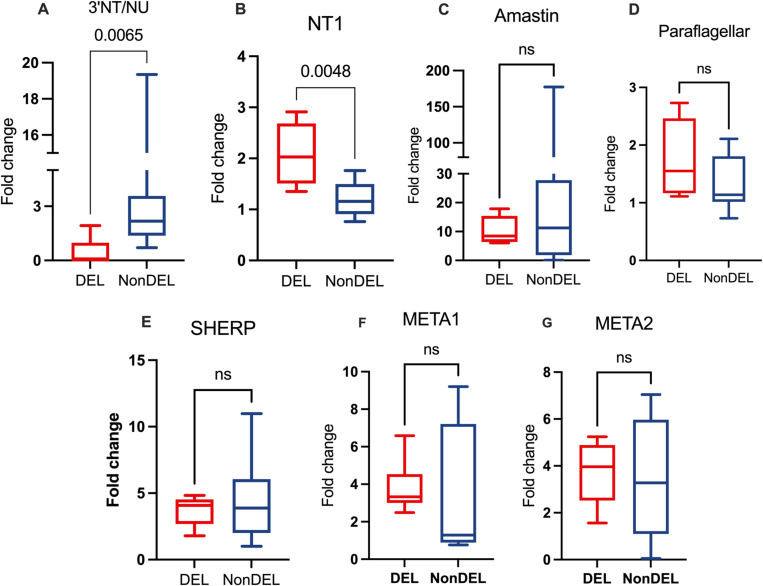
Fold change of transcripts from culture adapted promastigotes harvested from the late stationary phase (72 hours). The targets 3’NU/NT = 3’ecto-nucleotidase; NT1 = nucleoside transporter 1, Amastin and Paraflagellar Rod Protein were chosen based on previous data pointing to differences in CNV between DEL and NonDEL [4]. SHERP, META1 and META2 were included as potential markers for metacyclic [26] and for resistance to oxidative stress [27]. Total RNA was reversed transcribed in cDNA and targets quantified by Real Time qPCR. Delta-Delta Ct method was applied using alpha-tubulin as endogenous control and the OW sample NonDEL 3124 as calibrator. Mann Whitney U test (unpaired) for DEL (n = 5) and NonDEL (n = 9) samples. ns = not significant.

**Fig 3:**
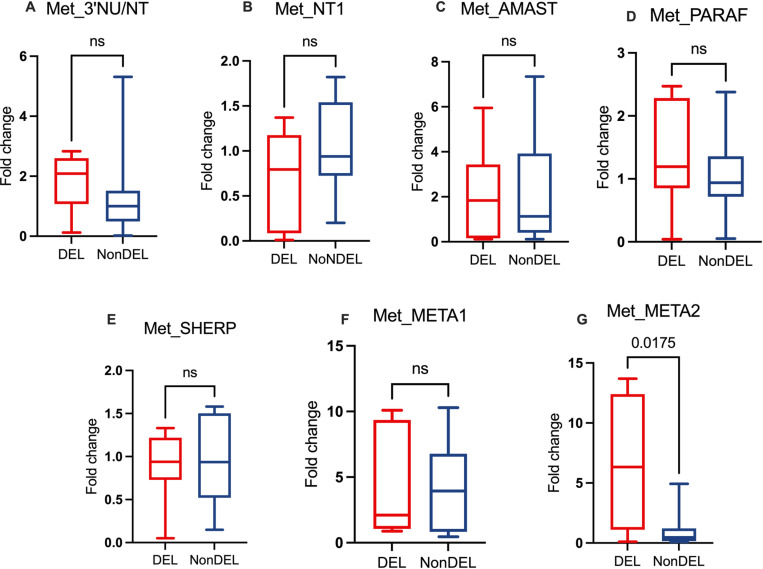
Fold change of transcripts from metacyclic-enriched cultures obtained by PNA from the late stationary phase (PNA−). The targets 3’NU/NT = 3’ecto-nucleotidase; NT1 = nucleoside transporter 1, Amastin and Paraflagellar were chosen based on previous data pointing to differences in CNV between DEL and NonDEL [4]. SHERP, META1 and META2 were included as potential markers for metacyclic and for resistance to oxidative stress [27]. Total RNA from both PNA+ and PNA− cultures were reversed transcribed in cDNA and targets quantified by Real Time qPCR. Delta-Delta Ct method was applied using alpha-tubulin as reference gene and the sample in PNA+ used as a calibrator for the correspondent sample in PNA− culture. Mann Whitney U Test (unpaired) for DEL (n = 6) and NonDEL (n = 9) samples. ns = not significant.

Our previous data [4] demonstrated among DEL strains higher gene copy number of the NT1 adenosine/pyrimidine nucleoside transporter gene (NT1), involved in the salvage route for purines by promoting the uptake of extracellular adenosine by the parasite [24]. It is reported that starvation of *Leishmania donovani* parasites for purines leads to a rapid amplification in purine nucleobase and NT [9]. Thus, the higher expression of this membrane transporter could represent a compensatory effect for the 3’NU/NT deficient parasites. Our data [4] also revealed reduced gene copy number for Paraflagellar and Amastin genes, which are associated with the parasite’s ability to infect mononuclear cells [25]. To assess whether these copy number variations (CNVs) are mirrored on transcript levels, we conducted relative quantitation using Real-Time qPCR under two conditions: i) culture-adapted promastigotes harvested at the late stationary phase (Fig 2), and ii) metacyclic-enriched parasites culture (PNA−; Fig 3). The results confirmed the decrease in transcripts for 3’NU/NT among DEL samples in stationary-phase promastigotes (Fig 2A), but not in PNA− parasites (Fig 3A). Additionally, the number of transcripts corroborated the CNV data for NT1 [4], with higher fold change in transcript level observed in the DEL group (2.0 vs 1.16 fold change median; Fig 2B). This suggests that alternative molecular pathways may emerge in mutant parasites, in accordance with report showing that starvation of *Leishmania donovani* for purines leads to a rapid amplification of transcripts for NT1 [9] gene. The results for Paraflagellar or Amastin transcripts (Fig 2C-D) did not align with previous CNV data, suggesting that a potential association between lower Paraflagellar and Amastin expression and reduced infectiousness in DEL samples is not supported.

SHERP, META1 and META2 were assayed as potential markers for metacyclogenesis [26] and for resistance to oxidative stress, as reported for *L. amazonensis* [27]. All presented similar transcript levels between groups. In PNA− samples, however (Fig 3), the DEL group exhibits higher fold change (6.6 vs 1.0; Fig 3F) for META2, suggesting these metacyclics may be less susceptible to oxidative stress and heat shock [27]. Although the META1 Fold Change did not differ significantly between genotype groups, the levels were higher in the metacyclic-enriched fraction compared to their paired PNA+ fraction (S3A Fig). This trend was observed when all samples (DEL and NonDEL) were analyzed together. However, when the groups were analyzed separately, the difference persisted only for NonDEL parasites, suggesting it as a potential metacyclic marker only for this *L. infantum* genotype. Curiously, META2 was under expressed for these same metacyclic-enriched fractions (S3B Fig). Therefore, although metacyclics did not differ quantitatively for META 1 and META 2 for DEL and NoNDEL, they differ qualitatively, i.e., PNA− differ from their respective PNA+ fraction (S3 Fig).

### DEL parasites show equal or higher fitness in key Brazilian vector species compared to NonDEL parasites

The adaptation of *L. infantum* parasites to different sand fly vectors involved in transmission in Brazil and the Mediterranean was evaluated using insect colonies. We included assays with *P. perniciosus*, an Old-World vector, as DEL strains have not yet been reported outside the Americas. We hypothesized that, despite the deficiency caused by the sub-chromosomal deletion, DEL parasites may be equally adapted—or even more efficient—at colonizing New World vectors compared to Old-World ones, when compared to NonDEL strains. Given the crucial role of sand flies in parasite transmission, this hypothesis offers a plausible explanation for the widespread distribution of DEL strains in Brazil and their restriction to the Americas. To test this, we measured various complementary parameters, including insect infection rates, parasite counts, stomodeal valve infections, and the detection of metacyclic forms.

**Fig 4:**
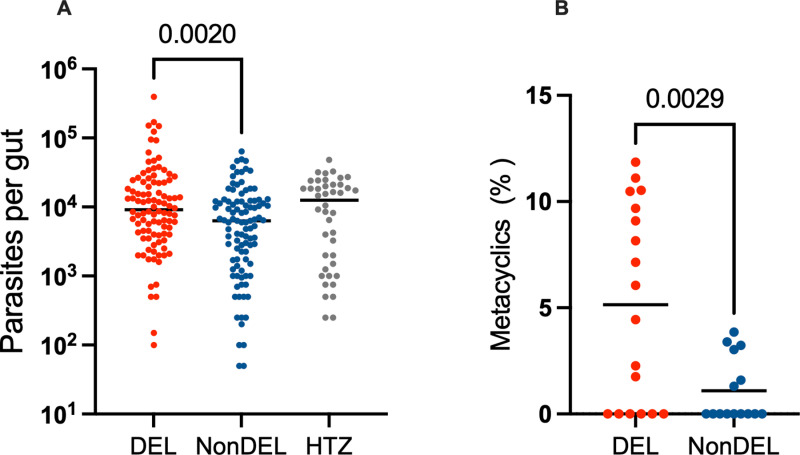
Infection outcome with DEL, Non-DEL and HTZ strains in L. longipalpis. (A) Number of Parasite per midgut. In red is represented the merged result of independent infection assays with three DEL strains (n = 3): Mato Grosso (MT_3223), Piauí (DEL_PI_2976) and Rio de Janeiro (DE_RJ_3598). In blue is represented the merged result of independent infection assays with three NonDEL strains (n = 3): MS_2666, PI_2972 and MT_3210. In grey is depicted the result obtained with one HTZ strain HTZ_MT_3134. (B) Percentage of metacyclic parasite forms detected in insect gut after infection with two DEL (n = 2; DEL_3223_MT, DEL_2976_PI) and two NonDEL (n = 2; NonDEL_3210_MT, NonDEL_2972_PI.) strains. All sand fly infection parameters were assessed at 192h (day 8) post-infection. Mann-Whitney test was used for pair-wise comparisons of parasite numbers expressed as the average of the biological replicates, and parasite forms.

First, we assessed the impact of the main vector for visceral leishmaniasis in Brazil, *L. longipalpis* [28], on the spread of DEL strains. Sand flies were infected with strains carrying the deletion from Rio de Janeiro (RJ), Mato Grosso (MT) and Piauí (PI), (DEL n = 3) and with NonDEL strains (n = 3) from Mato Grosso do Sul (MS), MT and PI, and one HTZ strain from MT (n = 1). Such geographic origins were considered to broadener the coverage of distinct biomes. After 192 hours, infection rates were similar, (S4A Fig) but DEL strains showed mean higher parasite loads per midgut (23939 parasites per gut) than NonDEL (9956), while HTZ (13182 parasites per gut) did not differ from neither group, indicating that the deletion potentially boosts fitness during the vector life cycle stage (Fig 4A). Moreover, metacyclics were quantified (DEL n = 2 and NoNDEL n = 2) and higher percentage of this infective forms were detected for the DEL group (mean 5.14% vs 1.09%, Fig 4B) suggesting higher transmissibility by the *L. longipalpis* for these parasites.

The individual analysis applied for each DEL and NonDEL strain selected for the vector infection assay confirmed the overall different outcomes between genotypes (S4B Fig), except for NonDEL 2972_PI. This sample presented similar parasite count to the three DEL strains (S4B Fig).

To assess the adaptation of parasites to different sand fly vectors we examined the impact of the sub-chromosomal deletion during infections assays in *L. migonei,* considered a secondary vector of leishmaniasis that possibly play a significant role in transmission, particularly in areas where the primary vector is less common. The assays revealed no differences in infection parameters, though a slight increase in stomodeal valve colonization and metacyclic form numbers was noted with the MT-DEL strain in *L. migonei* (S5A Fig). PI-DEL strain led to higher *L. migonei* sand flies infected and colonized stomodeal valve (76% vs 40% in NonDEL; 68.7% vs 31.25 in NonDEL, respectively) (S5B Fig). Results suggest that in regions where this vector is present, it may not favor the transmission of any given specific *Leishmania* genotype, equally contributing to the dispersion of DEL and NonDEL.

Infections with *P. perniciosus* were also performed to test whether different outcomes would occur when an Old-World sand fly vector of *L. infantum* was infected with American DEL and NonDEL strains. Interestingly, even in this species, DEL parasites presented higher or equivalent number of parasite count located at the stomodeal valve. One exceptional result was observed for sample DEL_PI_2976, which presented fewer metacyclic forms (S5C-D Fig). Overall, the deficient DEL parasites successfully infect, colonize, and undergo metacyclogenesis in the main Old and New-World vector species.

### The DEL parasites showed reduced ability to escape killing from neutrophil NETs and to infect macrophages and reduced or similar-to NonDEL ability to induce cellular recruitment in the mice model

**Fig 5:**
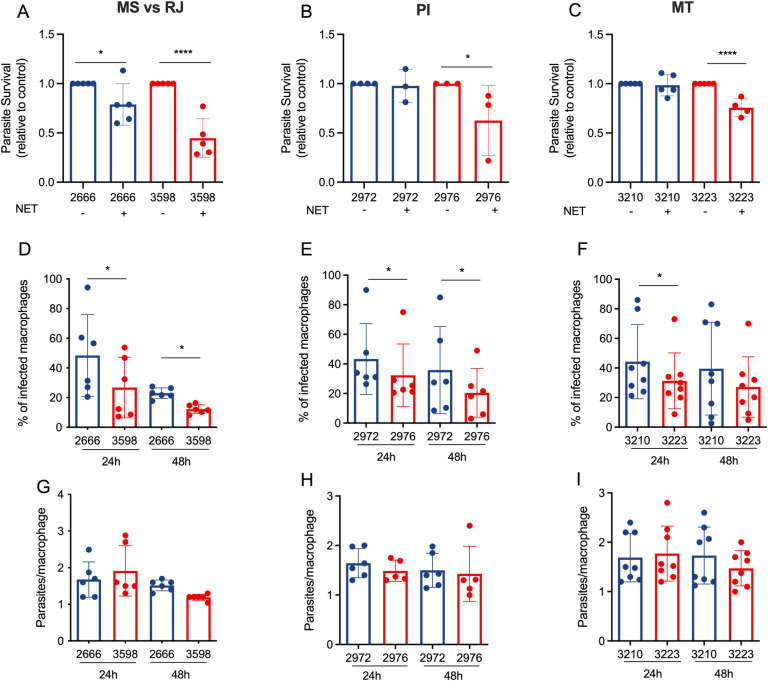
DEL parasites have a reduced ability to infect macrophages and escape killing from neutrophil NETs. (A-C) L. infantum metacyclics [5 × 10^5^] were incubated in the presence or absence of supernatants enriched in NETs for 4h at 35^o^C. Alamar blue (10% v/v) was added, and cells were incubated for more 4h at 35^o^C. Alamar blue fluorescence was read at 540/590 nm excitation/emission on a SpectraMax fluorimeter. Results are expressed as relative to control (parasites without NETs). Results of at least 3 independent experiments are shown as mean ± SEM. One-way ANOVA followed by Fisher’s LSD post-hoc comparison tests was performed. *p < 0.05 and ****p < 0.01. (D-F) Raw macrophages (2x10^5^) were seeded onto coverslips and then infected with Leishmania parasites at a cell ratio of 1 macrophage to 5 parasites. After 24h at 35^o^C, free parasites were washed out and coverslips were either fixed or incubated with medium (RPMI + 2.5% FBS) for more 24h (48h time point). Cells were stained with Panoptic dye kit and the number of infected macrophages (D-F) and the number of amastigotes/macrophage (G-I) were counted. Results are shown as mean ± SEM. Wilcoxon t-test was performed. *p < 0.05. Blue bars = NonDEL; red bars = DEL strains.

We performed a killing assay to compare the ability of DEL and NonDEL parasites to survive exposure to NETs. Results reveal that DEL metacyclic parasites are less resistant to NETs (44.6, 62.5 and 75.6% survival) than NonDEL (78.8, 97.7, and 93.7%), relative to their controls (Fig 5A-C). In the macrophage infection assay, the percentage of infected cells by DEL parasites was lower than that by the Non-DEL at 24 hours (26.8 vs 48.6%; 32.2 vs 43.3%; 31.4 vs 44.2%) and at 48 hours (20.4 vs 35.8%; 21.6 vs 35.4%; 27.1 vs 38.5%;) (Fig 5D-F). No difference in the number of parasites per macrophage was observed, (Fig 5G-I), suggesting differences in parasite virulence are more relevant in the initial stages of infection, and less evident after *Leishmania* achieves the intracellular stage.

The ecto-3’-nucleotidase activity, which is absent in DEL strains, is involved in the infection process of *Leishmania* in macrophages [11,13] and contributes to the escape of the parasite from neutrophil extracellular traps (NETs) [13,14]. Our results thus corroborate the literature describing the 3’NU/NT as a virulence factor relevant for the establishment of infection and reveal a reduced virulence of DEL parasites in vitro.

**Fig 6:**
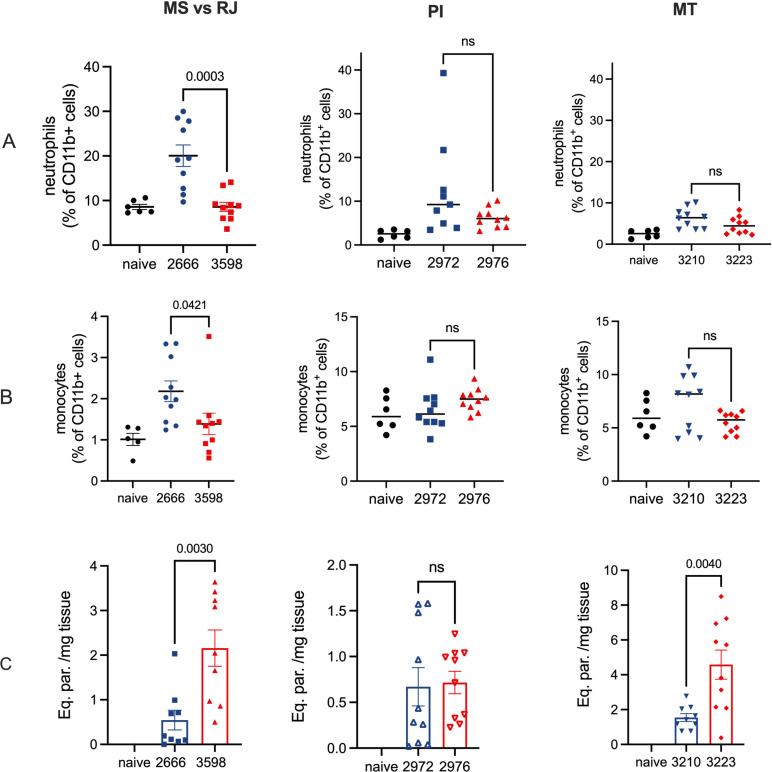
Neutrophil and monocyte recruitment to the site of infection by DEL and NonDEL parasites and parasite load from the draining lymph nodes. A - B) percentage of CD11b+ cells obtained from inoculated ear 12–15 hours PI (neutrophil and monocyte recruitment, respectively). Blue = NonDEL strains; Red = DEL strains. L. infantum metacyclic (10^5^) was inoculated in the inner ear of anesthetized BALB/c mice. 12–15 hours post-infection animals were euthanized, and ears were collected in RPMI. Ordinary one-way ANOVA. C) Parasite load expressed by equivalent parasite per mg of the draining lymph nodes from the ear. Material was collected and preserved in DNAShied for further DNA isolation and qPCR. Strains used in the assays were geographically unrelated: RJ = Rio de Janeiro; MS = Mato Grosso do Sul; MT = Mato Grosso; PI = Piauí.

The murine ear model assay was applied to assess the virulence of DEL/NonDEL strains from the previous experiments. Metacyclic parasites (10^5^) from passage-controlled cultures were inoculated intradermally at the inner ear and, 12–15 hours post-infection, neutrophil and monocyte recruitment were determined by flow cytometry. The data show reduced neutrophil and monocyte recruitment for DEL strains from the RJ vs MS pair (1.4 vs 2.1% of CD11b+ cells, respectively) (Fig 6A-B). The draining lymph nodes were harvested for parasite quantitation at the same time point (Fig 6C) and exhibited higher parasite load for the DEL strains from RJ (2.2 vs 0.5) and MT (4.6 vs 1.5 eq. par/mg tissue). This finding reflects these parasite’s ability to disseminate to distinct areas from that of the inoculated site, despite their increased susceptibility to NETs and macrophage defense. Moreover, similar ability of both genotypes to stablish visceral infection was confirmed by the detection of parasites in bone marrow (by qPCR), and in spleen and liver, by both qPCR and parasite isolation, at 3- and 5-weeks post intravenous infection (S6 Fig).

## Discussion

The occurrence of a 12Kb deletion on chromosome 31, restricted to American *L. infantum* strains, highly frequent and geographically widely spread [4], suggests this genomic trait conveys fitness for this population of parasites in New World conditions, despite hampering parasite’s important purine salvage pathways by depleting 3’NU/NT activity. Compensatory strategies and potential associated epidemiological consequences in the American Visceral Leishmaniasis (AVL) scenario due to the circulation of the distinct genotypes is a critical open question. On this ground, a first and pivotal action is to investigate the more prominent biological consequences of the deletion and assess its effect on parasite/ hosts interaction.

In this study, we utilized biological replicates of geographically unrelated mutant strains to better reflect the outcome of multiclonal and multi-genotype infections that occur in the field. Our aim was to explore the substantial differences between DEL and NonDEL *L. infantum* and infer about the intricate balance between fitness gain and cost in multi-host pathogenic parasites. However, a direct link between observed phenotypes and each of the genes within the deleted locus still requires verification by the development of knockout and add-back parasites.

Our in vitro data corroborates previous findings on the reduced or absent 3′ NT activity of geographically unrelated DEL promastigotes [4]. Furthermore, these DEL parasites exhibited, on average, higher rates of in vitro metacyclogenesis, likely triggered by nutritional stress resulting from the compromised purine salvage route dependent on 3’NU/NT activity [6]. A moderate to strong correlation was detected for the 3′ NT activity and metacyclogenesis parameters. Despite this evidence, we cannot exclude potential effects from the other genes lost on the metacyclogenesis.

The deficiency in 3’NT activity appears to induce a state akin to “starvation,” prompting an increase in the expression of NT1, a membrane transporter associated with purine uptake. This corroborates observations in *Leishmania donovani* parasites deprived of purines, in which rapid amplification in purine nucleobase and nucleoside transporter 1 (NT1) [9]was observed, suggesting this as an alternative adaptive compensatory mechanism. Further experimentation under controlled purine-deprived conditions would offer deeper insights into the biological behavior of DEL parasites. It’s also worth considering the composition of vector saliva as a potential molecular modulator of DEL and NonDEL strain behavior during blood feeding. Previous studies have reported the presence of AMP and purines (adenosine) in the saliva of *Phlebotomus papatasi e Phlebotomus duboscqi* sand fly, but, curiously, not in *L. longipalpis* [29]. These differences could influence the behavior and interactions of these parasites in each sand fly species. Delving into this aspect further could provide valuable insights into the transmission dynamics of DEL and NonDEL strains.

We further noted that the 3’NT activity in NonDEL is high in promastigotes, particularly in metacyclic forms. This implies that the enzyme may play an important role during the intravectorial phase or the early stages of mammalian infection. This raises the intriguing question of how DEL parasites, lacking 3’NU/NT, manage to overcome this enzyme deficiency and effectively infect hosts. It is conceivable that the higher rate of metacyclogenesis, negatively correlated with 3’NT activity, aids in overcoming this challenge and promotes successful infection. Additionally, our finding of higher transcripts of META2 in DEL metacyclic-enriched cultures suggests enhanced resistance to oxidative and heat shock stress [27] for the infective form of these strains.

The metacyclic promastigotes used in the in vitro experiments were obtained through peanut agglutinin (PNA) selection in culture. While this method does not fully replicate the metacyclic promastigotes isolated from the anterior thoracic midgut of sand flies [30], it provides a practical approach to compare various strains under standardized conditions. Future studies using metacyclic promastigotes obtained directly from in vivo sources would add value by enabling a more comprehensive comparison of the data.

In our experiments involving the main vector of *L. infantum* in the Americas, *Lutzomyia longipalpis*, DEL strains showed higher ability to colonize the insect’s midgut and increased metacyclogenesis. Increased parasite load in the vector midgut implies a higher dose of infective inoculum, that, combined to the presence of more infective forms boosts the odds of the parasite to establish infections [31,32] thereby promoting geographic spread. We also infected *L. migonei,* a species widely distributed and, although considered a secondary vector of *L. infantum*, can play a significant role in transmission, particularly in areas where the primary vector is less common. The present data did not show differences in the outcomes for DEL vs NonDEL, suggesting that in regions where this vector is present, it may not favor the transmission of any given specific *Leishmania* genotype, equally contributing to the dispersion of DEL and NonDEL.

These findings suggest that the deletion enhances fitness during the vector-borne life cycle stage in epidemiologically significant sand fly species. Thus, considering the pivotal role of *L. longipalpis* and *L. migonei* in the transmission cycle of AVL, it is probable that the interaction between DEL parasites and this vector species facilitates the spread of this genotype in the Americas. However, the extent of this phenomenon may vary depending on the geographical region and the specific population of *L. longipalpis* involved [33].

Contrary to the results of the sand fly experiments, the DEL parasites exhibited reduced fitness in the *in vitro* assays. This was evidenced by their lower virulence in macrophages and the diminished ability to escape killing by NETs. The results corroborate reports demonstrating that the downregulation of ecto-nuleotidases leads to decreased infectiousness [10] and lower number of surviving parasites following exposure to NETs [13]. This finding alone would suggest the DEL samples do not cause effective infection, but two evidences demonstrate the opposite. The first is the epidemiological data reporting a high prevalence of DEL parasites associated with human and canine cases of VL in the Americas [4,16]; the second is the presented result from the IV infection in mice confirming the ability of DEL to stablish visceral infection. The explanation for the paradox may rely on the mice ear early-infection model. The DEL strains induced reduced or similar-to NonDEL recruitment of neutrophil and monocyte, while presented higher parasite load in the draining lymph node. The reduced recruitment of neutrophils at the initial infection site leads to lesser effective control of the parasites, allowing them to migrate more quickly to the draining lymph node, thus facilitating the dissemination of the DEL parasites beyond the initial location. This represents an important strategy that may compensate DEL’s greater susceptibility to NETs and macrophages and allow the establishment of visceral infection.

The combined experimental findings suggest that NonDEL vs DEL metacyclic present important differences. The META2 gene expression, the different macrophage infection rate rather than different number of intracellular amastigotes, and the increased parasite number in the draining lymph node after ear inoculation indicate these metacyclic could have different infection competence. Thus, within the complex and not yet fully understood course of VL pathogenesis, it is plausible that mutant parasites cause effective, yet less pathogenic infection compared to NonDEL strains. Such variations in infectiousness and pathogenicity relate to host-parasite strategies of disease tolerance or resistance [34] that might have distinct evolutionary outcomes: resistance could reduce parasite prevalence, whereas tolerance could be neutral towards, or increase, prevalence in the population, ultimately reaching epidemiological consequences [34].

It is conceivable, thus, that the emergence of this potentially virulence-attenuating trait among New World *L. infantum* prompts disease tolerance/accommodation mechanisms within hosts, thereby favoring transmission throughout the DEL parasite’s life cycle [35]. Over time, such traits would likely undergo positive selection, especially if these mutant strains evolve strategies for long-term persistence within the host [36]. This scenario is better recognized in *Toxoplasma gondii* [37] in which increased tolerance by the host benefits the parasites, but it has not yet been fully elucidated in *Leishmania*.

Here we have described an adaptive gene loss in *L. infantum* strains that results in significant phenotypic variation, potentially impacting the expansion of the DEL mutant across the American continent. The DEL mutant strains, characterized by reduced pathogenicity, may exploit the trade-off between cost and benefit in immunity. They can infect and replicate, possibly without inducing exacerbated clinical signs that prompt treatment or culling of the main urban reservoir, the domestic dog, which is an undesirable consequence for parasite transmission. If this holds true, it strengthens the importance of detecting asymptomatic individuals as a policy to control the spread of disease. Animals without clinical signs would remain undetected for longer periods of time, representing a continuous source of infection for the sand flies in the area, favoring parasite spread [38,39]. Simultaneously, these DEL strains possess the evident ability to colonize the vector and undergo metacyclogenesis, generating an effective inoculum. Taken together, these attributes signify a gain in fitness at the population level and help elucidate the higher prevalence of deleted parasites in Brazil. This scenario, coupled with the observed natural resistance of DEL parasites to MIL, highlights the epidemiological and clinical significance of identifying the genotype of the infecting *L. infantum* in both animals and humans.

## Conclusion

The series of phenotypic variations we have demonstrated for DEL *L. infantum* parasites from Brazil may confer a net fitness gain across the parasite’s life cycle at the population level, representing an efficient mechanism for maintaining and dispersing the strains. The suggested mechanisms and implications for the circulation of DEL samples are not definitive but offer plausible explanations that warrant further investigation. Importantly, the results described, along with the different susceptibility to miltefosine, indicate that the genomic site of the deletion is a crucial molecular marker for clinicians and surveillance efforts, both to monitor the epidemiology of Visceral Leishmaniasis in the country and to further comprehend the consequences of the circulation of these distinct parasites.

## Methods

### Ethics statement

All L. infanum strains in this study were obtained from the Coleção de Leishmania da Fundação Oswaldo Cruz (CLIOC). In all cases Leishmania were isolated from human/canine patients as part of normal diagnosis and/or treatment with no unnecessary invasive procedures and with written and/or verbal consent recorded at the time of clinical examination. Balb/C mice were supplied by the Institute of Science and Technology in Biomodels (ICTB), Fiocruz and the experimental design was approved by Fiocruz Animal Welfare Committee (CEUA L-015/202-A2). All procedures involving human blood (for neutrophil NETs) were performed in accordance with the guidelines of the Research Ethics Committee (Hospital Universitário Clementino Fraga Filho, UFRJ, Brazil), protocol number: 4261015400005257.

### Parasites origin, maintenance and confirmation of species and genotype

All *L. infantum* strains sequenced in this study were obtained from the Coleção de Leishmania da Fundação Oswaldo Cruz (CLIOC). Associate information to the strains selected is depicted in S1 Fig. Strains were cultured in biphasic medium (Novy–MacNeal–Nicolle (NNN) + Schneider’s added Fetal Calf Serum 20%) prior to genomic DNA extraction (DNeasy Blood & Tissue Kit, Qiagen). Species were confirmed by multilocus enzyme electrophoresis (MLEE) as presented in the internal SOP. Real time qPCR protocol previously published [4] was applied to detect and/or confirm the deleted genotypes. Two different batches of cryopreservation were prepared: culture-adapted parasites (> than 20 passages), and passage-controlled parasites isolated from BALB/c mouse spleen and/or liver to be used in specific assays. Detailed information on the samples used in each experiment are presented in S1 Fig.

#### Metacyclic enrichment by PNA.

 Metacyclic enrichment was performed following protocols previously published [40]^.^ Briefly, parasites were washed twice in 50 ml Schneider (pure) by centrifuging 15 min at 3000 X g, 4C, in a 50-ml, resuspended in Schneider (pure), counted, and brought to 1–2x 10^8^/ml. The concentration of 0.01 vol of 5 to 10 mg/ml PNA was added to a final concentration of 50 to 100 μg/ml and parasites were incubate at room temperature for 15 to 30 min. After centrifugation for 5 min at 200 x g, 4C the collected supernatant was washed twice and resuspend in small volume of PBS for counting. The desired concentration can be obtained for inoculation preparation in PBS1x or for material to be preserved in TRiZOL for RNA isolation.

#### pH/temperature stressed culture (“axenic amastigote”).

 Promastigotes were transferred from the conventional culture condition (Shneider plus FCS 20%) to pure Schneider at pH6 and kept for 4 days at 37 °C. Morphology changes were monitored by light microscopy. In the fourth day, cells were subjected to 3’NT activity measurement.

#### 3’NT activity measurement.

The 3’-nucleotidase activity was determined as previously described [12,13] for NonDEL, HTZ and DEL strains. Briefly, the ecto-3’-nucleotidase activity was determined by measuring the rate of inorganic phosphate (Pi) production [11]. *L. infantum* parasites (2×10^7^ cells/mL) were incubated at 37 °C for 60 min in 0.5 mL of a reaction mixture containing 116mM NaCl, 5.4mM KCl, 5.5mM D-glucose, 50mM HEPES buffer (pH 7.4) and 1mM 3’-AMP as the substrate. Reactions were started by adding the cell suspension and stopped by centrifugation (1500 × g for 15 min), and 0.1 mL of the supernatant was added to 0.1 mL of Fiske-Subbarow reactive mixture to measure the Pi released into the supernatants at 650 nm. The concentration of Pi was determined using a standard curve for comparison. The ecto-3’-nucleotidase activity was calculated by subtracting the nonspecific 3’-AMP hydrolysis measured in the absence of cells. Experiments were performed at least twice, with similar results obtained from at least three separate cell suspensions. Values obtained for each strain was normalized by the ecto-3’-nucleotidase activity of *L. amazonensis* and unpaired t-test applied using PRIM 9. For the comparison between DEL, NonDEL and HTZ groups, the Mann-Whitney test was applied and *p<0.05 was considered as statistically different.

#### DNA isolation, standard curve and quantitative Real time PCR.

 Absolute quantitation of parasites in DNA isolated from lymph nodes was obtained by Standard curves. The protocol includes the TaqMan system (Applied Biosystems CA, USA) with mouse GAPDH (glyceraldehyde-3-phosphate dehydrogenase) as endogenous control (host target) (VIC/MGB probe) and the 18S rDNA (FAM/MGB probe) target for *Leishmania* (Fw 5’-GTACTGGGGCGTCAGAGGT-3’; 18S rDNA Rv 5’-TGGGTGTCATCGTTTGCAG-3’ and the probe 18S rDNA Tq 5’- FAM-AATTCTTAGACCGCACCAAG-NFQ-MGB-3’). The assay was performed, according to the following conditions: 95 °C for 10 min, 95 °C for 15 s and 59 °C for 1 min, 45 cycles carried out with Applied Biosystems VIAVII equipment available at Rede de Plataformas Tecnológicas Fiocruz (RPT09J). A DNA-free master mix as a No Template Control (NTC) and all samples and controls were performed in duplicate.

#### RNA extraction, cDNA synthesis and relative quantitation by Real Time qPCR.

Total RNA was isolated using TRIzol (Invitrogen) following the available protocol (Sigma-Aldrich). cDNA was synthetized with the SuperScript IV Synthesis Kit (Invitrogen) as recommended by the manufacturer. Both, total RNA and cDNA were normalized after quantification in NanoDrop 2.000 (Thermo Scientific). Relative quantitation of transcripts was done in a Quant Studio Real-Time equipment, available at Rede de Plataformas Tecnológicas Fiocruz (RPT09J). Reaction conditions and primers used were as follows: 0.3 micromolar of each primer and 1x Syber Green (Applied Biosystems). 15 sec at 95 °C; 1 min annealing and extension at 60 °C, for 40 cycles. Alpha-tubulin was used as endogenous control for the Delta-Delta Ct method. The NonDEL strain IOCL 3124, from Portugal, was used as a calibrator. Fold change comparison between DEL and NonDEL groups was performed by unpaired Mann Whitney t test in PRISM 9. *p < 0.05 was considered as statistically different. Primers used for the assays are presented in S1 Table.

#### Neutrophil purification.

Human neutrophils from the peripheral blood of healthy blood donors were isolated by density gradient centrifugation (Histopaque; Sigma–Aldrich) followed by hypotonic lysis of erythrocytes, as described previously [41].Purified neutrophils (≥95% of the cells) were resuspended in RPMI medium 1640 (Sigma). All experiments involving neutrophils were performed in RPMI.

#### Visualization of NETs.

Neutrophils (1 × 10^5^) were allowed to seed on 0.001% poly-L-lysine-coated coverslips and then incubated with promastigotes of *L. infantum* 2666 or 3598 (1 × 10^5^). After 3h, slides were fixed with 4% paraformaldehyde and stained with DAPI (10 ug/mL; Sigma) for 10 min. Images were taken on an EVOS FL Cell Imaging System.

#### Production of NETs-enriched supernatant and parasite survival assay.

 Neutrophils (8 × 10^6^) were activated with PMA (100 nM) for 4 h at 37 °C/5% CO_2_. Supernatants were recovered and the quantification of NETs was performed with the Picogreen dsDNA kit (Invitrogen, Life Technologies) as described [41]. *L. infantum* metacyclics [5 x 10^5^] were incubated in the presence or absence of supernatants enriched in NETs (500 ng/mL DNA) supplemented with 1%FBS for 4h at 35^o^C/5% CO_2_. Alamar blue fluorescence was read at 540/590 nm excitation/emission on a SpectraMax fluorimeter (Molecular Devices), using an excitation of 540 nm and emission of 600 nm. Data were normalized based on the fluorescence of control parasites cultured in the absence of NETs. Wilcoxon t-test analysis was performed and *p < 0.05 was considered as statistically different.

#### RAW 264.7 cell line infection.

 The RAW 264.7 murine macrophage cell line was maintained in RPMI 1640 media containing 10% heat-inactivated FBS. Raw macrophages (2 x 10^5^) were seeded onto coverslips and then infected with *Leishmania* parasites at a cell ratio of 1 macrophage to 5 parasites. After 24h at 35^o^C, free parasites were washed out and coverslips were either fixed or incubated with medium (RPMI + 2.5% FBS) for more 24h. Cells were stained with a Panoptic dye kit and the number of infected macrophages, and the number of amastigotes/macrophages were counted. Wilcoxon t-test analysis was performed and *p < 0.05 was considered statistically different.

#### Experimental infections of sand flies.

Experiments were performed using sand flies from a laboratory colony of *L. longipalpis* established from sand flies caught in Jacobina (Bahia, Brazil), *L. migonei* caught in Baturité (Ceará, Brazil), and *P. perniciosus* (Spain) using standard methods [42]. The insects were fed on 50% sucrose *ad libitum* and blood-fed on heparinized rabbit blood when needed. The insects were maintained at 27 ± 1 °C, humidity of 80–95%, and a photoperiod schedule of 12 h light/12 h dark. Three to five-day-old female sand flies were fed through chick skin membrane on heparinized rabbit blood containing 5 × 10^5^ *Leishmania* promastigotes/mL of each of the following strains at late log phase: IOCL 2666 (NonDEL), 3134 B1 (clone HTZ), IOCL 3598 (DEL), IOCL 3210 (non-DEL) and IOCL 3223 (DEL) isolated from Mato Grosso state (MT); and IOCL 2972 (non-DEL) and IOCL 2976 (DEL) isolated from Piauí state (PI). The midguts of insects were dissected 72 (day 3) and 192 hours (day 8) after infection. The number of infected sand flies was expressed as percentage of infected sand flies calculated in comparison of number of dissected guts. The number of parasites present per midgut was estimated from dissected guts homogenized in 50 μL of 0.85% NaCl + 1% formaldehyde solution, using a hemocytometer and plotted in log scale excluding zero values from non-infected sand flies. Localization of parasites in the sand fly gut was observed under 40x magnification [43] and expressed as percentage of sand flies that developed stomodeal valve stage infection in comparison to the number of dissected insects. Morphology of parasites was determined from 5 to 8 sand fly gut smears on glass slides stained with Giemsa, photographed under 100x magnification light microscopy, and measured using ImageJ software [44] and expressed as percentage of metacyclic promastigote forms in comparison to total number of analyzed parasite images from infected gut smears. A minimum of 3 independent experiments were preformed, and insects were analyzed individually. Statistical analysis was done using GraphPad Prism 6.07 computer software. Mann-Whitney test was used for pair wise comparisons of parasite numbers and the Chi-square test was used for contingency data of infected insects, infection localization, and parasite forms.

#### Ear model infection in BALB/c mice.

Female BALB/c mice were anesthetized with xylazine and ketamine. Using a 30G ultra-fine needle, 10 µL of 10^5^ metacyclic-enriched culture in PBS were inoculated intradermally in the inner part of both ears. 13–16 hours after inoculation animals were euthanized with xylazine and ketamine and ears were collected in RPMI on ice for flow cytometry. Draining lymph nodes were harvested and preserved in a lysis solution from High Pure DNA Isolation Kit (Roche).

#### Flow cytometry.

 Mice were euthanized, and the two sheets of ear dermis were isolated, placed in RPMI containing 0.2 μg/mL Liberase CI purified enzyme blend (Roche Diagnostics Corp.), and incubated for 1 h at 37 °C. After 1h, RPMI supplemented with FBS (10%) was added. Digested tissue was placed in a cell strainer (40 um; BD) and tissue debris were ground with the help of a syringe plunger. The resulting cells were stained with the Fixable Yellow Dead Cell Stain Kit (Invitrogen). Unspecific staining was blocked by incubating the ear cell suspension with mouse serum (10%). Cells were stained for Ly6C (BD Pharmingen, Cat #553104, Clone AL-21, Lot #7067529; 1:200; FITC), Ly6G (BD Pharmingen, Cat #551461, Clone 1A8, Lot #7068711; 1:800; PE), CD11b (BD Pharmingen™, Cat #552850, Clone M1/70, Lot #7033964; 1:800; PE-Cy7). Data were analyzed on a Fortessa flow cytometer (BD). Cells were acquired based on forward and side scatter, and live cells and data were analyzed with FlowJo Software 4.3.

## Supporting information

S1 Fig Details of parasites’ origin, maintenance, confirmation of species and genotype. **A)** The map depicts the geographic origin of the strains (except from Portugal strain) and reflects the wide distribution of the selected samples from Brazil. Base map data: OpenStreetMap contributors (available under the Open Database License, ODbL). **B)** The table shows *L. infantum* selected strains coded by CLIOC (Coleção de Leishmania da Fiocruz) voucher; international code; genotypes; geographic origin; host; and assays. Strains included in each assay are assigned “x”. Species (*L. infantum*) was confirmed by Multilocus Enzyme Electrophoresis (MLEE). Genotype was determined by Whole Genome Sequencing (WGS) and further confirmed by qPCR). Geographic origins: Northeast PE = Pernambuco, PI = Piaui, SE = Sergipe, BA = Bahia; Central-west: MT = Mato Grosso, MS = Mato Grosso do Sul; Southeast e South: RJ = Rio de Janeiro, SP = São Paulo, SC- Santa Catarina.
(DOCX)

S2 Fig Cell density and percentage of DEL and NonDEL metacyclic in two conditions.
**A)** Parasite density and **B)** percentage of metacyclic at stationary phase of culture with less than 20 passages (<20P) and adapted parasites with more than 30 passages (>30P). Percentage of metacyclic was determined 72 hours (late stationary phase) after an initial inoculum of 10∘6 parasites/ml. Metacyclic enrichment was obtained by Peanut agglutinin (PNA); percentage of cells from the PNA− fraction was determined in relation to the total cell count. Presence of metacyclic forms was confirmed by microscopy. Red = DEL; Blue=NonDEL; Unpaired t test.
(DOCX)

S3 Fig Fold change of transcripts from PNA+ and PNA− fractions at stationary phase.
The targets META1(A), META2 (B) and SHERP (C) were included as potential markers for metacyclic and for resistance to oxidative stress. Total RNA from both PNA+ and PNA− cultures were reversed transcribed in cDNA and targets quantified by Real Time qPCR. Delta-Delta Ct method was applied using alpha-tubulin as reference gene and the same sample in PNA+ used as a calibrator for the correspondent sample in PNA− culture (Fold Change = 1). In black: all samples, DEL and nonDEL combined; in red: only DEL samples; in blue: only NonDEL samples. T-test (paired) for DEL (n = 6 and 4) and NonDEL (n = 6 and 9) samples. ns = not significant. *P < 0.05; **P < 0.01.
(DOCX)

S4 Fig (A) Percentage of infected *L. longipalpis* and (B) parasite number detected in sandfly guts infected either with NonDEL_MT_3210, NonDEL_PI_2972 and NonDEL_MS_2666 (blue), HTZ_MT_3134 (grey), and DEL_MT_3223, DEL_PI_2976 and DEL_RJ_3598 (red) strains. Letters signal for statistical differences between groups (P < 0.05). (C) Percentage of insects that developed infection to the stomodeal valve, and (D) percentage of metacyclic parasite forms of NonDEL_MT_3210 and NonDEL_PI_2972 (blue) and DEL_MT_3223, DEL_PI_2976 (red) strains. Experiments performed at Fiocruz and Charles University colonies. All sand fly infection parameters were assessed at 192h (day 8) post-infection. Mann-Whitney test was used for pair-wise comparisons and the t-test. P values are presented when statically significant.
(DOCX)

S5 Fig Infections with DEL and Non-DEL strains from specific geographic areas in *L. migonei* and *P. perniciosus
* – Percentage of infected (A and B) *L. migonei* and (C and D) *P. perniciosus*, parasite number, percentage of insects that developed infection to the stomodeal valve, and percentage of metacyclic parasite forms of (A and B) NonDEL_MT_3210 (blue) and DEL_MT_3223 (red) strains from MT location, and (C and D) NonDEL_PI_2972 (blue) and DEL_PI_2976 (red) strains from PI location. All sand fly infection parameters were assessed at 192h (day 8) post infection. P values are presented when statically significant. Mann-Whitney test was used for pair-wise comparisons of parasite numbers expressed as the average of the biological replicates, and the Chi-square test was used for the sum of the biological replicates and expressed as contingency data of infected insects, infection localization, and parasite forms.
(DOCX)

S6 Fig Parasite load expressed by equivalent parasite per mg of tissue.
Animals were IV infected with 10∘7 metacyclic in the tail vain. Fragments of bone marrow, spleen, and liver of Balb/C mice were harvested 3 and 5 weeks after inoculum. Material was collected and preserved in DNAShield for further DNA isolation and qPCR. Unpaired t test. ns = Not significant. 2 independent experiments 4–5 animals per experiment.
(DOCX)

S1 Table Primers used for gene expression assays.
(DOCX)

S1 Data Excel file containing data used for statistics and figures.
(XLSX)
