# Correction: Gene deletion as a possible strategy adopted by New World *Leishmania infantum* to maximize geographic dispersion

**DOI:** 10.1371/journal.ppat.1013452

**Published:** 2025-08-28

**Authors:** Monique Florêncio, Marne Coimbra Batalha Chagas, Anderson Guimarães-Costa, Jullyanna Oliveira, Ingrid Waclawiak, Thamara K. F. Oliveira, Elvira Maria Saraiva, Anita Leocadio Freitas-Mesquita, José Roberto Meyer-Fernandes, Laura Aragão-Farias, Camilly Enes Trindade, Patricia Cuervo, Renata Azevedo do Nascimento, Otacilio C. Moreira, Flávia Lima Ribeiro-Gomes, Yara M. Traub-Csekö, Erich Loza Telleria, Slavica Vaselek, Tereza Leštinová, Petr Volf, Gerald F. Späth, Elisa Cupolillo, Mariana Côrtes Boité

There are errors in the Author Contributions. The correct contributions are:

**Conceptualization:** Gerald F. Späth, Elisa Cupolillo, Mariana Côrtes Boité

**Data curation:** Monique Florêncio, Marne Coimbra Batalha Chagas, Anderson Guimarães-Costa, Anita Leocadio Freitas-Mesquita, Erich Loza Telleria, Mariana Côrtes Boité

**Formal analysis:** Monique Florêncio, Anderson Guimarães-Costa, Anita Leocadio Freitas-Mesquita, Otacilio C. Moreira, Erich Loza Telleria, Mariana Côrtes Boité

**Funding acquisition:** Petr Volf, Gerald F. Späth, Elisa Cupolillo, Mariana Côrtes Boité

**Investigation:** Monique Florêncio, Marne Coimbra Batalha Chagas, Anderson Guimarães-Costa, Jullyanna Oliveira, Ingrid Waclawiak, Thamara K. F. Oliveira, Anita Leocadio Freitas-Mesquita, Laura Aragão-Farias, Camilly Enes Trindade, Renata Azevedo do Nascimento, Flávia Lima Ribeiro-Gomes, Erich Loza Telleria, Slavica Vaselek, Tereza Leštinová, Mariana Côrtes Boité

**Methodology:** Monique Florêncio, Marne Coimbra Batalha Chagas, Anderson Guimarães-Costa, Anita Leocadio Freitas-Mesquita,Jullyanna Oliveira, Ingrid Waclawiak, Thamara K. F. Oliveira, Laura Aragão-Farias, Camilly Enes Trindade, Otacilio C. Moreira, Flávia Lima Ribeiro-Gomes, Erich Loza Telleria, Slavica Vaselek, Tereza Leštinová, Petr Volf, Gerald F. Späth, Elisa Cupolillo, Mariana Côrtes Boité

**Project administration:** Mariana Côrtes Boité

**Resources** Elvira Maria Saraiva, José Roberto Meyer-Fernandes, Patricia Cuervo, Yara M. Traub-Csekö, Petr Volf, Elisa Cupolillo, Mariana Côrtes Boité

**Supervision:** Anderson Guimarães-Costa, Elvira Maria Saraiva, José Roberto Meyer-Fernandes, Patricia Cuervo, Yara M. Traub-Csekö, Erich Loza Telleria, Petr Volf, Elisa Cupolillo, Mariana Côrtes Boité

**Validation:** Marne Coimbra Batalha Chagas, Anderson Guimarães-Costa, Anita Leocadio Freitas-Mesquita, Erich Loza Telleria

**Visualization:** Monique Florêncio, Anderson Guimarães-Costa, Erich Loza Telleria, Mariana Côrtes Boité

**Writing – original draft:** Monique Florêncio, Mariana Côrtes Boité

**Writing – review & editing:** Anderson Guimarães-Costa, Elvira Maria Saraiva, Anita Leocadio Freitas-Mesquita, José Roberto Meyer-Fernandes, Patricia Cuervo, Otacilio C. Moreira, Flávia Lima Ribeiro-Gomes, Yara M. Traub-Csekö, Erich Loza Telleria, Petr Volf, Gerald F. Späth, Elisa Cupolillo, Mariana Côrtes Boité
