# Supplementary material for: Gene deletion as a possible strategy adopted by New World Leishmania infantum to maximize geographic dispersion
Source: PLoS Pathog. 2025 Mar 20;21(3):e1012938. doi: 10.1371/journal.ppat.1012938 (PMC11975383; doi:10.1371/journal.ppat.1012938)
Supplement: S1 Fig — Details of parasites’ origin, maintenance, confirmation of species and genotype. A) The map depicts the geographic origin of the strains (except from Portugal strain) and reflects the wide distribution of the selected samples from Brazil. Base map data: OpenStreetMap contributors (available under the Open Database License, ODbL). B) The table shows L. infantum selected strains coded by CLIOC (Coleção de Leishmania da Fiocruz) voucher; international code; genotypes; geographic origin; host; and assays. Strains included in each assay are assigned “x”. Species (L. infantum) was confirmed by Multilocus Enzyme Electrophoresis (MLEE). Genotype was determined by Whole Genome Sequencing (WGS) and further confirmed by qPCR). Geographic origins: Northeast PE = Pernambuco, PI = Piaui, SE = Sergipe, BA = Bahia; Central-west: MT = Mato Grosso, MS = Mato Grosso do Sul; Southeast e South: RJ = Rio de Janeiro, SP = São Paulo, SC- Santa Catarina. (DOCX) [file ppat.1012938.s001.docx]

A


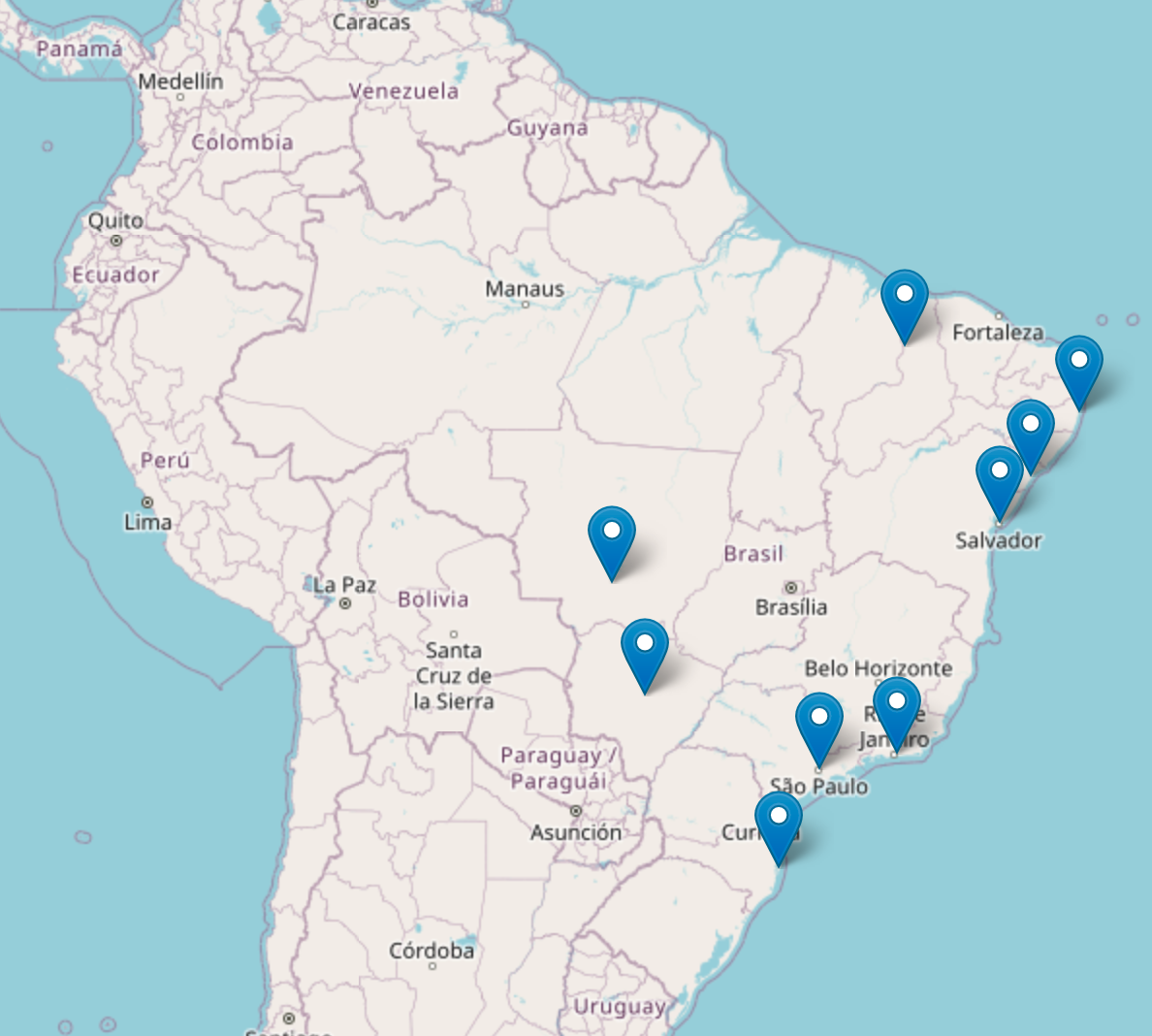


B

**S1 Fig**. **Details of parasites’ origin, maintenance, confirmation of species and genotype. A)** The map depicts the geographic origin of the strains (except from Portugal strain) and reflects the wide distribution of the selected samples from Brazil. Base map data: OpenStreetMap contributors (available under the Open Database License, ODbL). **B)** The table shows *L. infantum* selected strains coded by CLIOC (Coleção de Leishmania da Fiocruz) voucher; international code; genotypes; geographic origin; host; and assays. Strains included in each assay are assigned “x”. Species (*L. infantum*) was confirmed by Multilocus Enzyme Electrophoresis (MLEE). Genotype was determined by Whole Genome Sequencing (WGS) and further confirmed by qPCR). Geographic origins: Northeast PE= Pernambuco, PI=Piaui, SE= Sergipe, BA= Bahia; Central-west: MT=Mato Grosso, MS= Mato Grosso do Sul; Southeast e South: RJ= Rio de Janeiro, SP= São Paulo, SC- Santa Catarina.
