# Supplementary material for: Gene deletion as a possible strategy adopted by New World Leishmania infantum to maximize geographic dispersion
Source: PLoS Pathog. 2025 Mar 20;21(3):e1012938. doi: 10.1371/journal.ppat.1012938 (PMC11975383; doi:10.1371/journal.ppat.1012938)
Supplement: S2 Fig — A) Parasite density and B) percentage of metacyclic at stationary phase of culture with less than 20 passages (<20P) and adapted parasites with more than 30 passages (>30P). Percentage of metacyclic was determined 72 hours (late stationary phase) after an initial inoculum of 10∘6 parasites/ml. Metacyclic enrichment was obtained by Peanut agglutinin (PNA); percentage of cells from the PNA− fraction was determined in relation to the total cell count. Presence of metacyclic forms was confirmed by microscopy. Red = DEL; Blue=NonDEL; Unpaired t test. (DOCX) [file ppat.1012938.s002.docx]

**S2 Fig. Cell density and percentage of DEL and NonDEL metacyclic in two conditions. A)** Parasite density and B) percentage of metacyclic at stationary phase of culture with less than 20 passages (<20P) and adapted parasites with more than 30 passages (>30P). Percentage of metacyclic was determined 72 hours (late stationary phase) after an initial inoculum of 10ˆ6 parasites/ml. Metacyclic enrichment was obtained by Peanut agglutinin (PNA); percentage of cells from the PNA- fraction was determined in relation to the total cell count. Presence of metacyclic forms was confirmed by microscopy. Red = DEL; Blue=NonDEL; Unpaired t test.
