# Supplementary material for: Gene deletion as a possible strategy adopted by New World Leishmania infantum to maximize geographic dispersion
Source: PLoS Pathog. 2025 Mar 20;21(3):e1012938. doi: 10.1371/journal.ppat.1012938 (PMC11975383; doi:10.1371/journal.ppat.1012938)
Supplement: S3 Fig — The targets META1(A), META2 (B) and SHERP (C) were included as potential markers for metacyclic and for resistance to oxidative stress. Total RNA from both PNA+ and PNA− cultures were reversed transcribed in cDNA and targets quantified by Real Time qPCR. Delta-Delta Ct method was applied using alpha-tubulin as reference gene and the same sample in PNA+ used as a calibrator for the correspondent sample in PNA− culture (Fold Change = 1). In black: all samples, DEL and nonDEL combined; in red: only DEL samples; in blue: only NonDEL samples. T-test (paired) for DEL (n = 6 and 4) and NonDEL (n = 6 and 9) samples. ns = not significant. *P < 0.05; **P < 0.01. (DOCX) [file ppat.1012938.s003.docx]

**S3 Fig Fold change of transcripts from PNA+ and PNA- fractions at stationary phase.** The targets META1(A), META2 (B) and SHERP (C) were included as potential markers for metacyclic and for resistance to oxidative stress. Total RNA from both PNA+ and PNA- cultures were reversed transcribed in cDNA and targets quantified by Real Time qPCR. Delta-Delta Ct method was applied using alpha-tubulin as reference gene and the same sample in PNA+ used as a calibrator for the correspondent sample in PNA- culture (Fold Change = 1). In black: all samples, DEL and nonDEL combined; in red: only DEL samples; in blue: only NonDEL samples. T-test (paired) for DEL (n= 6 and 4) and NonDEL (n=6 and 9) samples. ns = not significant. *P<0.05; **P<0.01.
