# Supplementary material for: Gene deletion as a possible strategy adopted by New World Leishmania infantum to maximize geographic dispersion
Source: PLoS Pathog. 2025 Mar 20;21(3):e1012938. doi: 10.1371/journal.ppat.1012938 (PMC11975383; doi:10.1371/journal.ppat.1012938)
Supplement: S4 Fig — (A) Percentage of infected L. longipalpis and (B) parasite number detected in sandfly guts infected either with NonDEL_MT_3210, NonDEL_PI_2972 and NonDEL_MS_2666 (blue), HTZ_MT_3134 (grey), and DEL_MT_3223, DEL_PI_2976 and DEL_RJ_3598 (red) strains. Letters signal for statistical differences between groups (P < 0.05). (C) Percentage of insects that developed infection to the stomodeal valve, and (D) percentage of metacyclic parasite forms of NonDEL_MT_3210 and NonDEL_PI_2972 (blue) and DEL_MT_3223, DEL_PI_2976 (red) strains. Experiments performed at Fiocruz and Charles University colonies. All sand fly infection parameters were assessed at 192h (day 8) post-infection. Mann-Whitney test was used for pair-wise comparisons and the t-test. P values are presented when statically significant. (DOCX) [file ppat.1012938.s004.docx]

**S4 Fig**. (A) Percentage of infected *L. longipalpis* and (B) parasite number detected in sandfly guts infected either with NonDEL_MT_3210, NonDEL_PI_2972 and NonDEL_MS_2666 (blue), HTZ_MT_3134 (grey), and DEL_MT_3223, DEL_PI_2976 and DEL_RJ_3598 (red) strains. Letters signal for statistical differences between groups (P<0.05). (C) Percentage of insects that developed infection to the stomodeal valve, and (D) percentage of metacyclic parasite forms of NonDEL_MT_3210 and NonDEL_PI_2972 (blue) and DEL_MT_3223, DEL_PI_2976 (red) strains. Experiments performed at Fiocruz and Charles University colonies. All sand fly infection parameters were assessed at 192h (day 8) post-infection. Mann-Whitney test was used for pair-wise comparisons and the t-test. P values are presented when statically significant.
