# Supplementary material for: Gene deletion as a possible strategy adopted by New World Leishmania infantum to maximize geographic dispersion
Source: PLoS Pathog. 2025 Mar 20;21(3):e1012938. doi: 10.1371/journal.ppat.1012938 (PMC11975383; doi:10.1371/journal.ppat.1012938)
Supplement: S5 Fig — – Percentage of infected (A and B) L. migonei and (C and D) P. perniciosus, parasite number, percentage of insects that developed infection to the stomodeal valve, and percentage of metacyclic parasite forms of (A and B) NonDEL_MT_3210 (blue) and DEL_MT_3223 (red) strains from MT location, and (C and D) NonDEL_PI_2972 (blue) and DEL_PI_2976 (red) strains from PI location. All sand fly infection parameters were assessed at 192h (day 8) post infection. P values are presented when statically significant. Mann-Whitney test was used for pair-wise comparisons of parasite numbers expressed as the average of the biological replicates, and the Chi-square test was used for the sum of the biological replicates and expressed as contingency data of infected insects, infection localization, and parasite forms. (DOCX) [file ppat.1012938.s005.docx]

**S5 Fig. Infections with DEL and Non-DEL strains from specific geographic areas in *L. migonei* and *P. perniciosus*** – Percentage of infected (A and B) *L. migonei* and (C and D) *P. perniciosus*, parasite number, percentage of insects that developed infection to the stomodeal valve, and percentage of metacyclic parasite forms of (A and B) NonDEL_MT_3210 (blue) and DEL_MT_3223 (red) strains from MT location, and (C and D) NonDEL_PI_2972 (blue) and DEL_PI_2976 (red) strains from PI location. All sand fly infection parameters were assessed at 192h (day 8) post infection. P values are presented when statically significant. Mann-Whitney test was used for pair-wise comparisons of parasite numbers expressed as the average of the biological replicates, and the Chi-square test was used for the sum of the biological replicates and expressed as contingency data of infected insects, infection localization, and parasite forms.
