# Supplementary material for: Gene deletion as a possible strategy adopted by New World Leishmania infantum to maximize geographic dispersion
Source: PLoS Pathog. 2025 Mar 20;21(3):e1012938. doi: 10.1371/journal.ppat.1012938 (PMC11975383; doi:10.1371/journal.ppat.1012938)
Supplement: S6 Fig — Animals were IV infected with 10∘7 metacyclic in the tail vain. Fragments of bone marrow, spleen, and liver of Balb/C mice were harvested 3 and 5 weeks after inoculum. Material was collected and preserved in DNAShield for further DNA isolation and qPCR. Unpaired t test. ns = Not significant. 2 independent experiments 4–5 animals per experiment. (DOCX) [file ppat.1012938.s006.docx]

**S6 Fig. Parasite load expressed by equivalent parasite per mg of tissue.** Animals were IV infected with 10ˆ7 metacyclic in the tail vain. Fragments of bone marrow, spleen, and liver of Balb/C mice were harvested 3 and 5 weeks after inoculum. Material was collected and preserved in DNAShield for further DNA isolation and qPCR. Unpaired t test. ns=Not significant. 2 independent experiments 4-5 animals per experiment.
