# Supplementary material for: Gene deletion as a possible strategy adopted by New World Leishmania infantum to maximize geographic dispersion
Source: PLoS Pathog. 2025 Mar 20;21(3):e1012938. doi: 10.1371/journal.ppat.1012938 (PMC11975383; doi:10.1371/journal.ppat.1012938)
Supplement: S1 Table — (DOCX) [file ppat.1012938.s007.docx]

**Supp Table 1. Primers used for gene expression assays.**

| **Target** | **Primers** | **Primer design 3'-5'** |
| --- | --- | --- |
| *Nucleoside transporter 1 (LINF_150019900)* | NT1_F | CCGTGTCAAGTGGATGTTCG |
|  | NT1_R | TCGAGAACCACTTCGAGTCC |
| *Paraflagellar (LINF_050014400)* | Paraflag_F | TACAACTGTGACCTGGCGAT |
|  | Paraflag_R | AAGGTCCTGGTTCGTCTTGT |
| *Amastin (LINF_080011900)* | Amastin_F | ACGACCAGTGGAAGTTTTGC |
|  | Amastina_R | GCACAGCATAATGAAGCCGA |
| *Apha-tubulin (LINF_130007700)* | a-tubuln F | AGCACACCGATGTTGCGACGAT |
|  | a-tubulin R | GATCAGGCGGTTCACGTTCGTGT |
| *3'NT/NU (LINF_310031200)* | 31_2380_Fw_Tq | GCTGAAGTCAGTGAGCATGGA |
|  | 31_2380_Rv_Tq | TTCTGATCGTAGTGGTTGTGCA |
| *META1 (LINF_170016000)* | META1 F | CGAGAGTGGCGAACATCATG |
|  | META1 R | ATCCCTGACTGAGAGCGTTC |
| *META2 (LINF_170015800)* | META2 F | AACGGTGGAAGCTTTGTCAC |
|  | META2 R | CTGCTTCGCTCTGTGTTGTT |
| *SHERP (LINF_230018650)* | SHERP F | ACCAGGAGACAAAGGACCAG |
|  | SHERP R | GCCTTATTGCTCACACCGTC |
