# Supplementary material for: Gene deletion as a possible strategy adopted by New World Leishmania infantum to maximize geographic dispersion
Source: PLoS Pathog. 2025 Mar 20;21(3):e1012938. doi: 10.1371/journal.ppat.1012938 (PMC11975383; doi:10.1371/journal.ppat.1012938)
Supplement: Attachment — Submitted filename: Response_to_reviewers.docx [file ppat.1012938.s009.docx]

**Manuscript number:** RC-2024-02548R

**Corresponding author(s):** Mariana, Boité

*[Please use this template only if the submitted manuscript should be considered by the affiliate journal as a full revision in response to the points raised by the reviewers.*

*If you wish to submit a preliminary revision with a revision plan, please use our "*[*Revision Plan*](https://docs.google.com/document/u/0/d/1HHp62Tfeb44nd3Gu_3ChB0b8gmMdEog7GjZcZFwHSiQ/edit)*" template.* ***It is important to use the appropriate template to clearly inform the editors of your intentions.****]*

## 1. General Statements [optional]

We would like to thank Review Commons for providing this highly effective platform for peer-review process. The constructive comments and suggestions have significantly improved the manuscript, enhancing both its clarity and the depth of the discussion. We have accepted most of the suggestions and have modified the manuscript accordingly. Below we provide detailed answer to the reviewer’s comments.

Point-by-point description of the revisions

*This section is mandatory.* *Please insert a point-by-point reply describing the revisions that were already carried out and included in the transferred manuscript.*

**Reviewer #1**

***Evidence, reproducibility and clarity***

- *Florencio et al., presented a detailed analysis of Brazilian L. infantum isolates with about half with a 4 gene deletion on chromosome 31 and the other half without that deletion. This deletion was first observed in the context of miltefosine responsiveness by the Mottram group. Here a multinational team is concluding that this deletion is associated with increased metacyclogenesis, increase parasite load in some sandfly vector species factors that contribute to their prevalence in South American. Yet the deleted strains show decreased survival invitro including in macrophages, but from what I understand increased load in an animal model, possibly in line with their reduced efficacy in attracting/activating key immune cells.*

The authors thank for this clear overview.

- *The authors provide an interesting hypothesis on the equilibrium between pathogenesis (virulence) and capacity to transmit. The perfect parasite would like to infect as many hosts as possible while not killing them. I will try to provide the pro and cons.*
  *1. Obviously (and as acknowledged on p. 344-346) the conclusive experiment would be to add the 4 gene locus (the authors believe that it is the 3'NU/NT gene, so a single gene would even be simpler) in a DEL strain or alternatively KO the locus in a nonDEL strain. This would prove that the phenotype observed is indeed due to the loss of this locus. I understand that this is additional work but it would provide a definite answer. Now we have to rely on associations but alas the associations are not as tight as one would wish.*

We appreciate the reviewer’s valuable suggestion regarding the use of add-back parasites to directly link phenotypes with each of the deleted genes. This approach would indeed provide a definitive link between the deleted genes and phenotypes, and it is part of a separate project currently under development.

It is important to note that field isolates offer critical insights into the inherent nature of these parasites and contribute to a better understanding of eco-epidemiological dynamics, despite the challenges associated with working on such samples. In this manuscript, we aim to highlight the circulation of a distinct mutant *L. infantum* population and emphasize the significant biological differences observed among these Brazilian field strains, which have historically been considered quite homogeneous.

Given these goals, we believe the current approach aligns with the manuscript’s focus and would prefer to maintain it as presented.

- *Not sure if it is a dichotomy but they show decreased survival in the presence of NET (Fig. 6A) in vitro but reducing the attraction of immune cells including neutrophils in vivo (Fig. 7).*

At first may appear a dichotomy, but combined, these outcomes, along with the higher parasite load in dNF and ability to disseminate to the spleen, liver and bone marrow (as detailed in the new supplementary material added - Supp.Fig5), may reflect the parasites' strategy in effectively infecting despite reduced virulence, ultimately leading to fitness gains at the population level.

- *For the nonDEL strain they observe no effect of NET in vitro (Fig. 6) while they seem to attract more immune cells (Fig. 7) in vivo. The in vitro data seems to reach significance for the three group of strains but not for the in vivo work (one out of three).*

*The authors can be lauded for adding more pair of strains, but this also adds to the complexity and frankly raises questions. The two strains derived from the PI and MT location differs in many aspects compared to the MS-Rj comparators.*

Rationale Behind Adding More Strain Pairs: *Leishmania* field isolates can indeed vary significantly in many parameters. This variability is precisely why, when a consistent and stable set of traits—such as the deletion, lack of 3’NU/NT activity, and higher metacyclogenesis—is detected, the merge of strains as biological replicates represents a strategy to uncover additional common outcomes. Moreover, this approach more accurately reflects the complex, multiclonal, and multi-genotype infections that occur in the field.

While individual strains may not fully reveal a genotype-phenotype association, combining results from samples can highlight relevant common phenotypes that would otherwise remain undetected.

- *It is remarkable that the authors have tested other sandfly vector species (Fig. 5) but what is the conclusion and does it help in our understanding? I see variations in the figure between strains and vectors but not sure what it means.*

Thank you for your insightful comment. Based on the feedback from the reviewers (especially Reviewer 3, who noted that "The entire presentation of the sand fly infection experiments is confusing"), we have made significant changes to the presentation of the sand fly infection data. Below is a summary of these modifications:

- **Main Results in Figure 4:**
  We now present in the main document only the results of L. longipalpis infection assays, as this is the primary vector species for Leishmania infantum in Brazil. These results are particularly relevant to the propagation of DEL strains. Thus, the new Figure 4 exclusively displays parasite counts/metacyclic forms and combines data from independent infection assays involving three DEL, three NonDEL, and one HTZ strain. This approach better reflects the outcome of multiclonal and multi-genotype infections that occur in the field.
- **Supplementary Material Adjustments:**

The former Figure 4 has been moved to the Supplementary Materials as Supplementary Figure 3. Instead of the previous paired DEL vs. NonDEL analysis, we now present a full comparison of all DEL and NonDEL strains.

We consider this set of *L. longipalpis* data to suggest higher fitness for DEL strains in the primary vector species of *L. infantum* in Brazil, which likely contributes to the spread of the DEL genotype.

- **Secondary Vector Species (*Lutzomyia migonei* and *Phlebotomus perniciosus*):**

The previous Figure 5, which presented the results for *L. migonei* and *P. perniciosus*, has been moved to the Supplementary Materials as Supplementary Figure 4.

For *L. migonei*, although it is considered a secondary vector, it has a wide geographic distribution and plays a role in transmission, particularly where the primary vector is less common. Our data show no significant differences in outcomes between DEL and NonDEL strains, suggesting that in regions where *L. migonei* is present, it may not favor the transmission of any specific Leishmania genotype, contributing equally to the dispersion of both DEL and NonDEL strains.

Regarding *P. perniciosus*, the primary vector of *L. infantum* in the Mediterranean, it was noteworthy that DEL parasites also showed significant signals of fitness in this species, even though DEL *L. infantum* has only been recorded in the Americas. This finding raises interesting questions about vector compatibility across different regions.

**Conclusions from the Vector Assays:**
The data suggest that DEL parasites exhibit higher or equivalent fitness in the two primary Brazilian vector species compared to NonDEL parasites, which may contribute to the spread of the DEL genotype.

We have made corresponding revisions to the Results section and adapted the Discussion accordingly.

- *Secondary points
  1. Please confirm that the data in Fig. 1D and 1E are all the DEL and nonDEL strains. The number of dots does not match with the number of strains tested.*

Thank you for pointing this out.

We confirm that the data in **Fig. 1D** and **1E** include all the DEL and nonDEL strains. Specifically, we used a total of 7 DEL and 9 nonDEL strains to compare the average metacyclogenesis (updated Fig. 1D). For the paired t-test comparison of strains under two conditions (<20P and >30P), we used 6 DEL and 9 nonDEL strains (previously shown in **Figure 1F, G**, now updated as **Figure 1E, F**).

Based on your comment, we have moved the original **Figures 1D** and **1E** to the supplementary material (**Supp. Fig. 2**). The updated **Figure 1D** now presents the average of two experiments with the 7 DEL and 9 NonDEL strains. The text and figure legend have been revised accordingly.

Additionally, the table identifying each strain used in the experiments has been transferred to the supplementary material.

- *Do we really need Fig. 2 and 3? Not part of the main message.*

Thank you for this feedback. We believe that Fig. 2 and 3 are essential to the manuscript because they provide further evidence of the biological differences between the genotypes, as indicated by the relative gene expression. Furthermore, reviewer#3 provided valuable comments and suggestions on these figures, reinforcing their importance to our findings. Therefore, we would like to retain them in the manuscript.

- *Why is Fig. 4A only parasite count and 4B,C parasite count (please specify the number of hours) and additional information about stomodeal valve and metacyclics?*

Please, see the comments above regarding the sandfly’s experiments. We have modified significantly the presentation of the data.

Due to the large number of sand fly and *Leishmania* strains combinations and distinct infection parameters, we chose to assess such information on a single time point (192 hours). This information was added to the legend for clarity and is available at M&M.

- *4. Please explain sentence on p. 374-375. I thought that DEL is associated with increased metacyclics and increase parasite count in the sandfly.*

Thank you for this observation. Yes, our data indicate that DEL is associated with increased metacyclics and an increased parasite count in the sandfly. Yet, one might expect the higher proportion of the non-replicative metacyclic form would result in a lower overall parasite count. The recently described developmental form retroleptomonad (Serafim et al., 2018) however, suggests that one may find both, higher metacyclic and higher parasite count.

- *5. Just for curiosity, have the authors try to rescue the phenotype in vitro by adding purines in the medium with the DEL strains?*

Thank you for your interest in this aspect of our research. We are currently conducting experiments to explore this possibility. Preliminary results suggest that DEL strains respond differently to purine restriction in the culture medium, affecting both the cell cycle and metacyclogenesis.

**Reviewer #2**

***Evidence, reproducibility and clarity***

*In this manuscript the authors have investigated the role of sub chromosomal deletion found in new world Leishmania infantum species. The deletion (12 kb) spans over across the four copies of tetrasomic chromosome 31 which includes the loss of four open reading frames (ORF) LinJ.31.2370 (ecto-3ʹ-nucleotidase/nuclease), LinJ.31.2380 (ecto-3'-nucleotidase precursor), LinJ.31.2390 (helicase-like protein) and LinJ.31.2400 (3,2-trans-enoyl-CoA isomerase). The ecto-3'-nucleotidase/nuclease (3'NU/NT) activity has been shown to have an important role in trypanosomatids in the purine salvage pathway implicated as virulence factor affecting the parasite's ability to infect macrophages. The authors showed that having such deletions enhances the metacyclogenesis in vitro but are highly susceptible to killing by neutrophils and macrophages. In addition, they are also less virulent in vivo. The authors claim that enhanced metacyclogenesis increases its transmissibility in the invertebrate host but may not be highly infectious in the vertebrate host. They speculate that that such parasites may provide immune response that may control the infection in endemic population by having large group of asymptomatic individuals.*

- *These outcomes are highly speculative. This study is thought provoking but needs to be studied thoroughly.*

Thank you for the detailed summary of our findings. We acknowledge that the outcomes suggested for the circulation of DEL samples are not definitive; however, they represent plausible explanations that warrant further investigation.

As noted, parasites with the deletion—specifically lacking 3'NU activity—show enhanced metacyclogenesis and increased susceptibility to killing by neutrophil extracellular traps (NETs). This reinforces the well-established link between survival in NETs and 3'NU/NT activity. Furthermore, our previous research on the higher frequency and wider geographic distribution of DEL, which are restricted to the Americas, naturally leads to the propositions we have made.

Based on the reviewer's insightful comments, we have adjusted the conclusion to clarify these points, while maintaining them as potential hypotheses to be tested (please, see *Conclusion*).

*Following are the comments:*

- *1. Leishmania 3"NU/NT plays an important role in virulence of the parasite and its survival,
  a. how do 3'-NU/NT DEL parasites survive in the host which lack all the 4 copies of NT?*

Thank you for raising this important question. This is a major and fascinating topic we have been actively investigating. The question extends beyond how DEL parasites survive in the host; it also involves understanding how they manage to survive in different conditions and spread so effectively.

Leishmania parasites exhibit plasticity that leads to the development of alternative pathways at the genomic, transcriptomic, epigenetic, and proteomic levels. These alternative pathways may be influenced by selective pressures in New World environments. For example, a higher relative expression of the NT1 transporter has been observed in DEL strains, which primarily facilitates the uptake of adenosine from the extracellular environment into the parasites. This increased presence of NT1 in DEL strains may contribute to compensate for their reduced capacity to produce extracellular adenosine. To further understand these adaptative differences is our on-going aim.

- *b. what is the advantage of having such parasites circulating in the endemic areas?*

The circulation of DEL parasites in endemic areas may represent a population strategy for the parasite, allowing for efficient transmission and silent maintenance in reservoirs, such as dogs. This strategy may enable the parasites to spread more effectively and persist in the environment without causing overt disease, thereby facilitating their long-term survival and transmission within the host population. The reported association with reduced susceptibility to MIL contributes to the advantage for these DEL parasites to circulate, i.e., having parasites with low sensitivity will allow the parasite to persist in areas where miltefosine is being used, increasing the risk of transmission.

- *2. Since metacyclics are important for the pathogenesis of the parasites,
  a. What is the mechanism of increased metacyclogenesis of L. Infantum 3'-NU/NT DEL parasites in the absence of 3'-NU/NT activity which is essential for the virulence?*

One possibility is that the absence of 3'-NU/NT activity affects the parasite's ability to obtain purines, which are essential for replication. This purine deficiency could trigger metacyclogenesis as an adaptive response. We have preliminary data supporting this hypothesis; however, since it is part of an ongoing project, we have not included it in this manuscript.

- *3. How does lack of 3'NU/NT enhance transmissibility since such metacyclics from 3'-NU/NT DEL parasites barely survive in vivo?*

These metacyclics can survive in vivo, as evidenced by the frequent and widespread occurrence of DEL parasites (Schwabl et al., 2021) and the ability to stablish infection in mice (updated Supp.Fig5 shows parasites detected in spleen liver and bone marrow 3 and 5 weeks post infection). They likely persist at low but transmissible levels within the host, allowing them to be transmitted to sandflies over time. Thus, even with reduced survival in certain conditions, their ability to spread remains effective.

- *a. the parasites are less resistant to NET and are killed easily.*

Yes, but they undoubtedly survive enough to stablish infection (please, see Schwabl et al., 2021 and the data added to supp material Fig5 regarding mice IV infection. DEL parasites remained detectable in bone marrow, spleen and liver by qPCR 3-5 weeks PI and were successfully isolated and cultured 3 weeks PI from spleen).

- *b. there is reduced recruitment of neutrophils (NT) and monocytes in the ear.
  4. Fig.7C: what is the reason for higher parasite load of DEL in dLN?*

The increased parasite load in the draining lymph node (dLN) may indicate a greater ability to disseminate beyond the initial infection site. The observed reduced recruitment of neutrophils and monocytes may contribute to this dissemination. This suggests that the parasites might be evading the local immune response and spreading to other body parts. Therefore, although DEL parasites are more susceptible to neutrophil extracellular traps (NETs) and macrophages, the reduced recruitment of neutrophils and monocytes would allow them to spread beyond the initial infection site.

Additional observation: Studies with *Leishmania major* suggest that a higher parasite load in the dLN may indicate potential for chronic infection (Nicolas et al., 2000).

- *5. Do you think there is reduced recruitment of NT in the infected site which would have controlled the parasites, hence they migrate quickly in the dLN?*

Yes, this is a likely possibility, as discussed in our previous comments. Reduced recruitment of neutrophils at the initial infection site may lead to less effective control of the parasites, allowing them to migrate more quickly to the draining lymph node (dLN). This reduced immune response at the infection site could facilitate the dissemination of the parasites beyond the initial location, an important strategy for the fragile DEL parasite to stablish visceral infection. However, further discussion on the outcome would need specific cytokine data.

Based on the reviewer’s comment, we included the following sentence in the Text:“*The draining lymph nodes were harvested for parasite quantitation at the same time point exhibiting higher parasite load for the DEL strains from RJ (2.2 vs 0.5) and MT (4.6 vs 1.5 eq. par/mg tissue) suggesting a strategy to disseminate, despite their increased susceptibility to NETs and macrophage defense. The similar ability of both genotypes to stablish visceral infection was confirmed after intravenous inoculum by the detection of parasites in bone marrow (by qPCR), and in spleen and liver, by both qPCR and parasite isolation, at 3- and 5-weeks PI (Supp. Material, Supp. Fig5)*.”

- *To test this possibility the authors should perform an* *in vitro NT recruitment assay.*

Thank you for this suggestion. We plan to extend our assays in future studies to include an in vitro neutrophil recruitment assay. However, for the current manuscript, we will focus on the experiments already reported.

- *6. Line 417: How are the 3'-NU/NT DEL parasites continuous source for infection in sand flies, If such parasites are not infectious and will be cleared by the host?*

The 3'-NU/NT DEL parasites are certainly infectious; they form more metacyclics and are not completely cleared by the host. Evidence include: the high frequency of DEL parasites in most Brazilian regions (Schwabl et al. (2021); our unpublished surveillance data indicating that these parasites are frequently found in clinical skin samples from dogs; the IV experimental infection in Balb/C mice (Supp. Fig.5)

- *7. Line 400: Is it possible that having 3'-NU/NT DEL parasites in circulation dampens the infectivity of the NON-DEL and thus over all infection rate in the population goes down?*

If the DEL parasites are indeed less pathogenic combining strategies to spread efficiently, we would rather expect an overall increase in infection rate, but a reduction in symptomatic and severe outcomes.

While we do not know precisely how long these DEL parasites have been circulating in Brazil, our previous study (Schwabl et al., 2021) suggests they were introduced approximately 500 years ago and have since been either positively selected or have spread widely due to allele surfing. During this timespan, the Americas have experienced an increase in the incidence and geographic expansion of visceral leishmaniasis. However, it is challenging to draw definitive conclusions about the impact of DEL parasites on the overall infection rate.

- *8. Could the 3'-NU/NT DEL parasites be the source of asymptomatic infections?*

Yes, it is possible that 3'-NU/NT DEL parasites contribute to asymptomatic infections. We are currently investigating this hypothesis by comparing the clinical profiles of naturally infected dogs vs the *L. infantum* genotype. However, the numerous variables involved in natural infections, particularly in domestic dogs, introduce a higher level of uncertainty to this research that we aim to overcome.

- *9. Is there literature evidence for such a possibility in the endemic region?*

Not yet. The deletion was first described by Carnielli et all, 2018. This a current goal from our group.

**Reviewer #3**

***(Evidence, reproducibility and clarity (Required)):***

***Significance***

- *The authors claim that enhanced metacyclogenesis increases transmissibility of Leishmania in the invertebrate host but may not be highly infectious in the vertebrate host. They speculate that that such parasites may provide immune response that may control the infection in endemic population by having large group of asymptomatic individuals. These outcomes are highly speculative. This study is thought provoking but needs to be studied thoroughly.*

We thank the reviewer for the comments.

We respectfully disagree that these conclusions are speculative. Instead, we propose these outcomes as the best explanation based on the evidence gathered, which is a common methodological rationale used to discuss data.

Our manuscript raises these potential outcomes because they provide the best explanation for the evidence presented: the widespread geographic distribution and frequent occurrence of DEL parasites, higher metacyclogenesis, and the ability to infect vertebrate hosts with high parasite loads in the draining lymph node (dLN), despite lower susceptibility to NETs and macrophages.

Based on the concerns raised by the reviewer, we have adjusted the conclusion, while maintaining the outcomes as potential hypotheses to be tested (please, see *Conclusion*).

- *In this study, the authors investigate potential developmental changes in Leishmania infantum isolates from different regions of Brazil (plus a single isolate from Portugal) both in vitro and in vivo. The manuscript present interesting phenotypic comparisons between isolates with a 12-kb deletion of a subtelomeric region on Chr31 and isolates with a complete sequence. The authors suggest that distinctions associated with the presence or absence of that region may potentially be linked with fitness in the wild. A more detailed analysis of such valuable sample cohort would increase the significance of this research.*

*Therefore, I highlight a few important concerns that need to be addressed, as well as a disconnect between the presented data and conclusions made.*

- *Major issues -The % of metacyclics in all the sand fly infection experiments was extremely low ( <6%). The only exception was NonDEL_PI_2972, which showed around 16% metacyclics. This tells me that the parasites did not develop properly inside any of the 3 vectors used. Does an increase from 2% to 4% make any difference in vector competence? If metacyclogenesis is not significantly higher in DEL isolates, then there will most likely not be an increase in fitness concerning transmission. Also, I assume that both the stomodeal valve infection and the metacyclic % were assessed at 192h p.i., but this must be clearly stated in the text. At what time-point were parasites per gut counted at Charles Univ.? The parameters used in all the different sand fly experiments should be clearly labeled.*

Based on our extensive experience with various *Leishmania*-sand fly pairs, we conclude that the parasites developed relatively well across all combinations. The relatively low proportion of metacyclic promastigotes observed in gut smears of *Lutzomyia longipalpis* at 192 hours (day 8) could be explained by several facts:

1. Sampling location: Smears were taken from the entire gut, rather than specifically from the anterior (thoracic) region where metacyclic stages typically accumulate.
2. Timing: It is well-documented that the percentage of metacyclic promastigotes increases with time post-infection (e.g., Freitas et al. 2012). Therefore, it is likely that the proportion of metacyclic forms would be higher on days 10 and 12. However, due to the fragile nature of *L. migonei*, the experiments had to be terminated on day 8.
3. Comparison with previous studies: The percentages observed in this study are consistent with those reported in our earlier work on *L. infantum/donovani* (see below).

In this study, we did not test whether a 2% or 4% presence of metacyclic forms in the insect gut significantly impacts vector competence. However, our data indicated that the Del strain from MT developed twice as many metacyclic forms as the non-Del strain in *L. longipalpis* (the dat anow in Supp material). This difference could be crucial for vector competence. Using a similar method, Sadlova et al. (2017) detected less than 5% *Leishmania donovani* metacyclic promastigotes in *Phlebotomus argentipes*, and the same group of *P. argentipes* infected females had a transmission efficiency of 42% to mice. Therefore, we believe that even small differences, such as those between 2% and 4%, may lead to variations in transmission rates. All sand fly infection parameters assessed at Charles University (insect infection rates, parasite count, stomodeal valve infection, and metacyclic detection) were done at 192h (day 8) post-infection. This information was added to the results description and legends.

- *If the metacyclic % data are available for the Fiocruz colony experiments, then it will be important to present those. Conversely, are the #s of parasites/gut available using another time point at Charles Univ.?*

At Fiocruz, no morphometric analyses were performed. At Charles University, due to the large number of sand fly and *Leishmania* strains combinations and distinct infection parameters, we chose to assess such information on a single time point.

- *It is mentioned that at least 3 independent sand fly infections were performed, so the data points should be shown for each of the replicates where applicable. A bar plot without error bars is not ideal for representing the stomodeal valve and metacyclic % data either. Based on the current plots, it is difficult to infer biological significance.*

The column graphs represent contingency data; the sum of all experiments. Thus, the error bar does not apply to this data format. We rephrased it within the legend for clarification as follows:

“Mann-Whitney test was used for pair-wise comparisons of parasite numbers expressed as the average of the biological replicates, and the Chi-square test was used for the sum of the biological replicates and expressed as contingency data of infected insects, infection localization, and parasite forms.”

- *The entire presentation of the sand fly infection experiments is confusing and does not provide consistent findings to support the conclusions, which should be toned down.*

Thank you for your comments. While we respectfully disagree with the assertion that our data does not support the conclusions, we acknowledge that the presentation of the sand fly infection experiments may have been unclear and contributed to this impression.

To address this, we have made significant improvements to the clarity of the data, which are now reflected in the revised Figure 4, the Supplementary Material Fig.3-4 and adjustment in results and discussion sections. Please also see our responses to Reviewer #1.

- *The whole rationale for the different vector species should be better explained.*

**Rationale for the different vector species:** *Lutzomyia longipalpis* is the primary vector of *L. infantum* in the New World, while two other proven (secondary) vectors in Brazil are *Lutzomyia migonei* and *Lutzomyia cruzi*. Currently, there is no available colony of *L. cruzi* anywhere in the world, though a colony of *L. migonei* has been established in Prague.

Genotype distribution in Brazil shows widespread occurrence of DEL samples, with hot-spot areas of nonDEL strains. This led us to question whether specific transmission cycles involving distinct vector species might favor one genotype over the other in certain regions. Furthermore, since no DEL strains have been reported outside the Americas, the inclusion of the Old-World species *Phlebotomus perniciosus* in our assays allowed us to assess whether the mutation in *L. infantum* would result in better adaptation to New World sand flies.

The results indicate that DEL parasites either equally infect and colonize all sand fly species tested or show higher fitness, as observed in *L. longipalpis.*

- *The 3'NT activity in two HTZ isolates is as high as in some NonDEL, while in one HTZ strain it is as low as the DEL isolates. Is there any genomic difference between the HTZ to justify this difference? Since 3254 HTZ was not included in the qPCR analysis presented in Schwabl, et al. 2021, it is difficult to see any correlation.*

We investigated potential genomic differences but found no variations that could explain the observed differences in 3'NT activity among the HTZ isolates. The variability in the enzyme activity is likely due to post-transcriptional and transcriptional regulatory mechanisms. There are also significant differences within the NonDEL group, despite the fact there is no variation in genomic copy number among these strains.

- *Pg. 16 ln.398: The authors conclude that DEL isolates cause effective, yet less pathogenic infection compared to NonDEL isolates. However, it is not possible to reach that conclusion based on the early-infection data presented.*

The main evidence that supports the affirmation that the DEL infection is effective is the higher frequency and widespread distribution of these parasites in Brazil, detected among the assessed *Leishmania* samples from our biobank (Schwabl et al., 2021) and from clinical samples of naturally infected animals and humans (unpublished).

- *For that, i.v.-infected mice would have to be monitored for several weeks and assessed for visceralization and VL severity.*

Based on the relevant reviewer’s comments, we decided to include additional evidence in supplementary material (Supp.Fig 5) providing the data of parasite loads from Balb/C IV infected mice with the DEL 3598 and NoNDEL 2666 strains. Spleen, liver, and bone marrow were harvested at 3- and 5-weeks post-infection. Parasites were successfully isolated despite the low parasite load detected by qPCR for both strains. We added this comment to the text.

- *The most relevant data in this work suggest that metacyclics in nonDEL vs DEL might present important differences. The META2 gene expression, the different M0 infection% rather than different # of intracellular amastigotes, and increases in parasite #s in the draining lymph node at 13-16 hours after ear inoculation suggest to me that the metacyclics could have different infection competence. That possibility should be addressed.*

Thank you very much for this accurate observation on the combined data. We included that comment in the discussion.

- *Do they display similar morphology? Do they differentiate at similar rates to axenic amastigotes?*

We did not perform controlled analytical assays to evaluate the differentiation rates to axenic amastigotes or metacyclic morphology. We did prepare slides with parasites from PNA- fraction to confirm the efficacy of the PNA protocol, but they were not used for detailed morphological analysis.

- *SHERP transcript levels should be quantified in metacyclics from the different isolates.*

We have quantified SHERP transcript level as suggested, and the data was included in Figures 2 and 3.

*Minor issues*

- *Gene IDs of the transcripts measured should be listed somewhere.*

Thank you. The information was included in supp. Material.

- *Fig.1C, Fig2A&B: missing p-value labels.*

Thank you, we incorporated the values and included “ns” for the 3124 pro vs 3124 PNA- comparison.

- *Fig.1B needs more detail on the statistical test used. Is it One-way ANOVA? What post-hoc test was used?*

Thank you for pointing this out. The information was mistakenly presented. ANOVA was initially considered; however, not all data passed the normality tests (particularly in the NonDEL group). As a result, we applied the Kruskal-Wallis test, followed by pairwise comparisons using the Mann-Whitney U test. We have now included this information in the figure legend.

- *Figs. 2 & 3: "Mann Whitney Rank Sum", "Mann Whitney t-test". The name of the statistical test is either Mann Whitney U test or Wilcoxon rank-sum test.*

Thank you for pointing this out. The text was modified to Mann-Whitney U test in both figures.

- *Figs. 4 & 5: information missing on statistical tests used in both.*
- *Pg.8 ln.212: The NT1 median FC in the NonDEL group does not seem to be 0.8 in Fig.2B.*

Thank you very much for detecting that. We revised the data and indeed the correct value to 1.16. This was a mistake when transferring the medians values to the text.

*-Typos: 
-Fig.2 title: "promastigotes harvest from" -> harvested*

Done *-Fig2. legend: "Paraflagellar". Is this a paraflagellar rod protein (PFR)? If so, it should be specified throughout the text.*

Done *-Fig 2 & 3: "ns = no significant" -> not significant*

Done *-Different parts of the text: "alfa-tubulin" -> alpha-tubulin*

Done *-Fig.4A: Y-axis reads "Inspected sand flies"* 
