# Supplementary material for: Gene deletion as a possible strategy adopted by New World Leishmania infantum to maximize geographic dispersion
Source: PLoS Pathog. 2025 Mar 20;21(3):e1012938. doi: 10.1371/journal.ppat.1012938 (PMC11975383; doi:10.1371/journal.ppat.1012938)
Supplement: Attachment — Submitted filename: Response to Reviewers.docx [file ppat.1012938.s010.docx]

**Responses do Reviewers.**

***Reviewers' Comments:***

*Reviewer's Responses to Questions*

***Part I – Summary***

*Please use this section to discuss strengths/weaknesses of study, novelty/significance, general execution and scholarship.*

***Reviewer #1****:*

*1) The authors have clarified all the points concerning sand fly infections and improved the data visualizations.*

We thank the valuable comments and questions made by Reviewer #1. They contributed to the discussion and enriched the manuscript.

*2) The use of PNA to purify L. infantum metacyclics remains a topic of debate. It would be useful to include images of both PNA- and PNA+ parasites to support findings. Alternatively, direct readers to relevant literature that demonstrates enrichment of L. infantum metacyclics in the PNA- population (e.g., Soares et al 2002; Alcolea et al 2016).*

Thank you for raising this important point. Indeed, as demonstrated by Alcolea et al., 2016, the PNA- population does not fully replicate the metacyclic promastigotes isolated from the anterior thoracic midgut of sand flies. However, this limitation is shared by many other in vitro experimental approaches and the use of axenic parasites. Despite this, the PNA-based protocol offers a practical and reliable method to compare a representative set of strains, making it a valuable tool for this study.

We have added the Alcolea et al., 2016 reference to **Lines 434-439**

1) *It remains to be confirmed if the DEL genotype is linked to parasite attenuation, as there were no attempts to engineer transfectants to reconstitute the NonDEL phenotype. However, I appreciate that such genetics studies are not trivial and may be beyond the scope of the current paper.*

Thank you for the comment. Please, see below the answer we presented to a similar query made by the editor:

As noted by Reviewer #1, conducting assays with engineered transfectants is not a trivial task, and the reviewer agreed that such experiments represent a separate study that would require full completion and publication. This aligns with our plans for future research.

That said, we would like to share with the editor our latest results with recently obtained addback parasites. We successfully generated addback lineages for the first and second deleted copies of the 3′-nucleotidase/nuclease (Addback 200 and Addback 300, respectively—the latter identified as the functional copy of 3′-nucleotidase/nuclease by Carnielli et al., 2020). We then repeated the 3′-ectonucleotidase activity assay and the metacyclic enrichment experiments, and the preliminary data further support the hypothesis under investigation. **As addressed before, these are the first results we have from a different study we will submit as a separate manuscript.**

Enzymatic Activity:

- In the late stationary phase, the enzymatic activity of WT DEL (red) and NonDEL (blue) was consistent with our previous results.
- Addback 300 significantly restored enzymatic activity compared to WT DEL but did not achieve the higher levels observed among WT NonDEL.

Cell Density & Metacyclic Enrichment:

- Similar to previous observations, NonDEL samples reached higher cell densities at the late stationary phase.
- WT DEL and WT NonDEL reproduced earlier findings for metacyclic enrichment, with WT DEL showing higher percentages.
- The Addbacks exhibited an intermediate profile, with Addback 300 again differing significantly from DEL and NonDEL.

We agree that a full study with transfectants would be highly valuable for understanding the role of each gene in the observed outcomes. As stated above, we are currently conducting a separate study involving transfectants that will complement the data presented here. However, we also believe that the use of field parasites in biological replicates (different strains) provides valuable information on these differences without the added complexities of transfectants. Together, both studies will provide a comprehensive understanding of the phenomenon.

*4) I recommend that the following limitations in the current work should also be made clear in the Discussion.*

*(a) The BALB/c iv infections did not show significant decrease in parasite loads in the DEL strain vs the NonDEL strain in the bone marrow, spleen or liver. This suggests a similar pattern of dissemination between DEL and NonDEL strains.*

Thank you for raising this point. Indeed, our data supports the claim that, despite reduced infectivity in vitro, DEL parasites successfully establish visceral infections during the early stages of in vivo infection. Once the parasites reach this stage, their deficiencies do not hinder their ability to disseminate and replicate intracellularly in target organs similarly to NonDEL.

*Of note, no pathology measure was provided in those infections (e.g., splenomegaly), which would have been valuable to assess experimental disease severity.*

We agree with the reviewer that a detailed study on the pathology of both genotypes would be highly relevant. We have the material prepared for histopathological analysis in a separate study. However, our current study specially aims to show that, despite their deficiencies in infecting cells in vitro, DEL strains effectively appear in the draining lymph node after ear inoculation and are equally capable of infecting and replicating in the spleen and liver following intravenous inoculation.

*(b) Although other findings seem to indicate changes in the infection competence of the purified metacyclics, the unchanged expression of all but one of the metacyclic markers between DEL and NonDEL suggests that there is little apparent intrinsic differences between the metacyclic populations.*

In the submitted version of the manuscript, we plotted the mean differences between DEL and NonDEL fold change. Based on comments from the editor, we have now included **Supp. Fig3** and refer to it at **line 244-252**, depicting that although DEL and NonDEL metacyclics only differ on META2 (on average), when we paired the PNA+ vs. PNA- values of fold change, META1 appears more expressed in NonDEL PNA- compared to the corresponding PNA+ fraction (SuppFig3A), but not for the DEL samples. This approach also showed that the higher mean of META2 for DEL is due to its underexpression in NonDEL samples rather than overexpression in DEL (SuppFig3B). Despite further interpretations that may arise from these finding, it reinforces that DEL and NonDEL metacyclics are indeed different.

*(c) At this stage, it is uncertain whether the DEL strains truly ‘possess a remarkable ability to colonize the vector and undergo metacyclogenesis’, in the context of more mature infections in prior work using Lu. longipalpis-Linf combinations (e.g., Rogers et al 2010; Giraud et al 2019). Additionally, an association with ‘higher transmissibility by Lu. longipalpis’ has yet to be confirmed.*

We agree with the reviewer that an association with *Lu. longipalpis* has yet to be confirmed, which could include add-back experiments, molecular approaches, and feeding models with infected insects. However, these studies are beyond the aim of the current manuscript. To address this, we have modified the tone of the claim by withdrawing the word "remarkable." In **line 502**, we changed the phrase to “Simultaneously, these DEL strains possess the evident ability to colonize the vector..."

***Reviewer #2:***

*The authors have responded and made changes in the conclusions. However, some of the experiments which were suggested by the reviewers they were not able to do, which they claim are part of the ongoing studies.*

Thank you for the comment. Yes, we address that to the editor as well.

*Given the fact that it is novel finding that L. infantum species lacking the 3NUT gene has resulted in the reduction in infectivity and increase transmissibility. The authors have speculated that such parasites may cause asymptomatic infections and could provide immunity without causing the disease. That needs to be seen in future studies.*

*With this in mind I would suggest that the paper may be accepted.*

We thank the reviewer for this positive response and for all valuable contributions made to the manuscript.

***Part II – Major Issues: Key Experiments Required for Acceptance*** *Please use this section to detail the key new experiments or modifications of existing experiments that should be absolutely required to validate study conclusions.

Generally, there should be no more than 3 such required experiments or major modifications for a "Major Revision" recommendation. If more than 3 experiments are necessary to validate the study conclusions, then you are encouraged to recommend "Reject".*

*Reviewer #1: None*

*Reviewer #2: (No Response)*

***Part III – Minor Issues: Editorial and Data Presentation Modifications*** *Please use this section for editorial suggestions as well as relatively minor modifications of existing data that would enhance clarity.*

*Reviewer #1:*

**Typos* Supp. Fig. 5 (bone marrow panel): ‘semanas’ -> weeks Fig.4 not referenced in the text (Error)*

Thank you for detecting that. The error was corrected.

*Reviewer #2: None*
