# Supplementary material for: Gene deletion as a possible strategy adopted by New World Leishmania infantum to maximize geographic dispersion
Source: PLoS Pathog. 2025 Mar 20;21(3):e1012938. doi: 10.1371/journal.ppat.1012938 (PMC11975383; doi:10.1371/journal.ppat.1012938)
Supplement: Peer review history

# Author comment: Author response to Decision Letter 0

**DOI:** 10.1371/journal.ppat.1012938.r001

1 Nov 2024

Attachment Submitted filename: Response_to_reviewers.docx

# Peer review: Decision Letter 0

**DOI:** 10.1371/journal.ppat.1012938.r002

**Authors:** Margaret A Phillips, Dawn M Wetzel

17 Dec 2024

PPATHOGENS-D-24-02287

Gene deletion as a possible strategy adopted by New World Leishmania infantum to maximize geographic dispersion

PLOS Pathogens

Dear Dr. Boité,

Thank you for submitting your manuscript to PLOS Pathogens. After careful consideration, we feel that it has merit but does not fully meet PLOS Pathogens's publication criteria as it currently stands. Therefore, we invite you to submit a revised version of the manuscript that addresses the points raised during the review process.

Please submit your revised manuscript within 60 days Feb 15 2025 11:59PM. If you will need more time than this to complete your revisions, please reply to this message or contact the journal office at plospathogens@plos.org. When you're ready to submit your revision, log on to https://www.editorialmanager.com/ppathogens/ and select the 'Submissions Needing Revision' folder to locate your manuscript file.

Please include the following items when submitting your revised manuscript:

* A rebuttal letter that responds to each point raised by the editor and reviewer(s). You should upload this letter as a separate file labeled 'Response to Reviewers'. This file does not need to include responses to any formatting updates and technical items listed in the 'Journal Requirements' section below.

* A marked-up copy of your manuscript that highlights changes made to the original version. You should upload this as a separate file labeled 'Revised Manuscript with Track Changes'.

* An unmarked version of your revised paper without tracked changes. You should upload this as a separate file labeled 'Manuscript'.

If you would like to make changes to your financial disclosure, competing interests statement, or data availability statement, please make these updates within the submission form at the time of resubmission. Guidelines for resubmitting your figure files are available below the reviewer comments at the end of this letter.

We look forward to receiving your revised manuscript.

Kind regards,

Dawn M Wetzel, MD, PhD

Academic Editor

PLOS Pathogens

Margaret Phillips

Section Editor

PLOS Pathogens

 Sumita Bhaduri-McIntosh

Editor-in-Chief

PLOS Pathogens

orcid.org/0000-0003-2946-9497

Michael Malim

Editor-in-Chief

PLOS Pathogens

orcid.org/0000-0002-7699-2064

**Additional Editor Comments:**

The authors have effectively revised their manuscript from an original submission to Review Commons to address prior reviewer comments. Two of the reviewers have commented on the revised manuscript below. The 3rd was not available.

Several deficiencies remain from the prior reviews, including:

1) One ongoing concern that remains from the unavailable reviewer is that there could be other unknown factors that could explain the differences seen in Del vs non-DEL strains. This concern would be mitigated through several approaches. Please improve the discussion of any statistics or analysis used to ensure that it was the lack of 3NU/NT activity in the clinical isolates that was responsible for reduced infectivity and increased transmissibility and not another factor related to the other deleted genes. Were any other changes among the DEL vs non-DEL strain groups noted in prior manuscripts or analysis that might affect conclusions here? In addition - if parasites with higher and lower levels of SHERP, META1 or META2 are grouped together instead of high vs low 3NU/NT activity, are there any differences seen in metacyclogenesis? Finally, the authors should make clear in the discussion that another, more conclusive means to show that it is specifically the lack of 3NU/NT activity that results in reduced infectivity and increased transmissibility would be through use of genetics in the same Leishmania background strain - eg, by introducing the locus/gene of interest in their DEL strain OR removing that locus from a non DEL strain - and explain why this was not done and how it will be done in the future.

2) Please otherwise ensure that conclusions match the data provided and do not overstate them, as discussed by the reviewers.

3) Please address the other concerns below from the 2 reviewers.

**Journal Requirements:**

1) Please provide an Author Summary. This should appear in your manuscript between the Abstract (if applicable) and the Introduction, and should be 150-200 words long. The aim should be to make your findings accessible to a wide audience that includes both scientists and non-scientists. Sample summaries can be found on our website under Submission Guidelines:

https://journals.plos.org/plospathogens/s/submission-guidelines#loc-parts-of-a-submission

2) We do not publish any copyright or trademark symbols that usually accompany proprietary names, eg ©,  ®, or TM  (e.g. next to drug or reagent names). Therefore please remove all instances of trademark/copyright symbols throughout the text, including:

- ® on Lines: 516 and 548.

3) Please upload all main figures as separate Figure files in .tif or .eps format. For more information about how to convert and format your figure files please see our guidelines:

https://journals.plos.org/plospathogens/s/figures

4) Please upload a copy of Figure 8 which you refer to in your text on page 19. Or, if the figure is no longer to be included as part of the submission please remove all reference to it within the text.

5) We have noticed that you have uploaded Supporting Information files, but you have not included a list of legends. Please add a full list of legends for your Supporting Information files after the references list.

6) Some material included in your submission may be copyrighted. According to PLOSu2019s copyright policy, authors who use figures or other material (e.g., graphics, clipart, maps) from another author or copyright holder must demonstrate or obtain permission to publish this material under the Creative Commons Attribution 4.0 International (CC BY 4.0) License used by PLOS journals. Please closely review the details of PLOSu2019s copyright requirements here: PLOS Licenses and Copyright. If you need to request permissions from a copyright holder, you may use PLOS's Copyright Content Permission form.

Please respond directly to this email and provide any known details concerning your material's license terms and permissions required for reuse, even if you have not yet obtained copyright permissions or are unsure of your material's copyright compatibility. Once you have responded and addressed all other outstanding technical requirements, you may resubmit your manuscript within Editorial Manager.

Potential Copyright Issues:

- Figure S1; Please provide a direct link to the base layer of the map (i.e., the country or region border shape) and ensure this is also included in the figure legend; and provide a link to the terms of use / license information for the base layer image or shapefile. We cannot publish proprietary or copyrighted maps (e.g. Google Maps, Mapquest) and the terms of use for your map base layer must be compatible with our CC BY 4.0 license.

Note: if you created the map in a software program like R or ArcGIS, please locate and indicate the source of the basemap shapefile onto which data has been plotted.

If your map was obtained from a copyrighted source please amend the figure so that the base map used is from an openly available source. Alternatively, please provide explicit written permission from the copyright holder granting you the right to publish the material under our CC BY 4.0 license.

If you are unsure whether you can use a map or not, please do reach out and we will be able to help you. The following websites are good examples of where you can source open access or public domain maps:

* U.S. Geological Survey (USGS) - All maps are in the public domain. (http://www.usgs.gov)

* PlaniGlobe - All maps are published under a Creative Commons license so please cite u201cPlaniGlobe, http://www.planiglobe.com, CC BY 2.0u201d in the image credit after the caption. (http://www.planiglobe.com/?lang=enl)

* Natural Earth - All maps are public domain. (http://www.naturalearthdata.com/about/terms-of-use/).

7) Please ensure that the funders and grant numbers match between the Financial Disclosure field and the Funding Information tab in your submission form. Note that the funders must be provided in the same order in both places as well. State the initials, alongside each funding source, of each author to receive each grant. For example: "This work was supported by the National Institutes of Health (####### to AM; ###### to CJ) and the National Science Foundation (###### to AM)." State what role the funders took in the study. If the funders had no role in your study, please state: "The funders had no role in study design, data collection and analysis, decision to publish, or preparation of the manuscript.".

If you did not receive any funding for this study, please simply state: u201cThe authors received no specific funding for this work.u201d

**Reviewers' Comments:**

Reviewer's Responses to Questions

**Part I - Summary**

Please use this section to discuss strengths/weaknesses of study, novelty/significance, general execution and scholarship.

Reviewer #1: 1) The authors have clarified all the points concerning sand fly infections and improved the data visualizations.

2) The use of PNA to purify L. infantum metacyclics remains a topic of debate. It would be useful to include images of both PNA- and PNA+ parasites to support findings. Alternatively, direct readers to relevant literature that demonstrates enrichment of L. infantum metacyclics in the PNA- population (e.g., Soares et al 2002; Alcolea et al 2016).

3) It remains to be confirmed if the DEL genotype is linked to parasite attenuation, as there were no attempts to engineer transfectants to reconstitute the NonDEL phenotype. However, I appreciate that such genetics studies are not trivial and may be beyond the scope of the current paper.

4) I recommend that the following limitations in the current work should also be made clear in the Discussion.

(a) The BALB/c iv infections did not show significant decrease in parasite loads in the DEL strain vs the NonDEL strain in the bone marrow, spleen or liver. This suggests a similar pattern of dissemination between DEL and NonDEL strains. Of note, no pathology measure was provided in those infections (e.g., splenomegaly), which would have been valuable to assess experimental disease severity.

(b) Although other findings seem to indicate changes in the infection competence of the purified metacyclics, the unchanged expression of all but one of the metacyclic markers between DEL and NonDEL suggests that there is little apparent intrinsic differences between the metacyclic populations.

(c) At this stage, it is uncertain whether the DEL strains truly ‘possess a remarkable ability to colonize the vector and undergo metacyclogenesis’, in the context of more mature infections in prior work using Lu. longipalpis-Linf combinations (e.g., Rogers et al 2010; Giraud et al 2019). Additionally, an association with ‘higher transmissibility by Lu. longipalpis’ has yet to be confirmed.

Lastly, although I acknowledge that some sections of the manuscript do have a more speculative tone, I must clarify that the ‘Significance’ paragraph included in the #3 section of ‘Response to Reviewers’ was not submitted by me.

Reviewer #2: The authors have responded and made changes in the conclusions. However, some of the experiments which were suggested by the reviewers they were not able to do, which they claim are part of the ongoing studies.

Given the fact that it is novel finding that L. infantum species lacking the 3NUT gene has resulted in the reduction in infectivity and increase transmissibility. The authors have speculated that such parasites may cause asymptomatic infections and could provide immunity without causing the disease. That needs to be seen in future studies.

With this in mind I would suggest that the paper may be accepted.

**********

**Part II – Major Issues: Key Experiments Required for Acceptance**

Please use this section to detail the key new experiments or modifications of existing experiments that should be absolutely required to validate study conclusions.

Generally, there should be no more than 3 such required experiments or major modifications for a "Major Revision" recommendation. If more than 3 experiments are necessary to validate the study conclusions, then you are encouraged to recommend "Reject".

Reviewer #1: None

Reviewer #2: (No Response)

**********

**Part III – Minor Issues: Editorial and Data Presentation Modifications**

Please use this section for editorial suggestions as well as relatively minor modifications of existing data that would enhance clarity.

Reviewer #1: *Typos*

Supp. Fig. 5 (bone marrow panel): ‘semanas’ -> weeks

Fig.4 not referenced in the text (Error)

Reviewer #2: None

**********

PLOS authors have the option to publish the peer review history of their article (what does this mean? ). If published, this will include your full peer review and any attached files.

If you choose “no”, your identity will remain anonymous but your review may still be made public.

**Do you want your identity to be public for this peer review?** For information about this choice, including consent withdrawal, please see our Privacy Policy .

Reviewer #1: No

Reviewer #2: No

[NOTE: If reviewer comments were submitted as an attachment file, they will be attached to this email and accessible via the submission site. Please log into your account, locate the manuscript record, and check for the action link "View Attachments". If this link does not appear, there are no attachment files.]

**Figure resubmission:**

While revising your submission, please upload your figure files to the Preflight Analysis and Conversion Engine (PACE) digital diagnostic tool, https://pacev2.apexcovantage.com/. PACE helps ensure that figures meet PLOS requirements. To use PACE, you must first register as a user. Registration is free. Then, login and navigate to the UPLOAD tab, where you will find detailed instructions on how to use the tool. If you encounter any issues or have any questions when using PACE, please email PLOS at figures@plos.org. Please note that Supporting Information files do not need this step. If there are other versions of figure files still present in your submission file inventory at resubmission, please replace them with the PACE-processed versions.

**Reproducibility:**

To enhance the reproducibility of your results, we recommend that authors of applicable studies deposit laboratory protocols in protocols.io, where a protocol can be assigned its own identifier (DOI) such that it can be cited independently in the future. Additionally, PLOS ONE offers an option to publish peer-reviewed clinical study protocols. Read more information on sharing protocols at https://plos.org/protocols?utm_medium=editorial-email&utm_source=authorletters&utm_campaign=protocols

# Author comment: Author response to Decision Letter 1

**DOI:** 10.1371/journal.ppat.1012938.r003

23 Jan 2025

Attachment Submitted filename: Response to Reviewers.docx

# Editor report: Decision Letter 1

**DOI:** 10.1371/journal.ppat.1012938.r004

**Authors:** Margaret A Phillips, Dawn M Wetzel

27 Jan 2025

Dear Mrs Boité,

We are pleased to inform you that your manuscript 'Gene deletion as a possible strategy adopted by New World Leishmania infantum to maximize geographic dispersion' has been provisionally accepted for publication in PLOS Pathogens.

Before your manuscript can be formally accepted you will need to complete some formatting changes, which you will receive in a follow up email. A member of our team will be in touch with a set of requests.

Please note that your manuscript will not be scheduled for publication until you have made the required changes, so a swift response is appreciated.

IMPORTANT: The editorial review process is now complete. PLOS will only permit corrections to spelling, formatting or significant scientific errors from this point onwards. Requests for major changes, or any which affect the scientific understanding of your work, will cause delays to the publication date of your manuscript.

Should you, your institution's press office or the journal office choose to press release your paper, you will automatically be opted out of early publication. We ask that you notify us now if you or your institution is planning to press release the article. All press must be co-ordinated with PLOS.

Thank you again for supporting Open Access publishing; we are looking forward to publishing your work in PLOS Pathogens.

Best regards,

Dawn M Wetzel, MD, PhD

Academic Editor

PLOS Pathogens

Margaret Phillips

Section Editor

PLOS Pathogens

Sumita Bhaduri-McIntosh

Editor-in-Chief

PLOS Pathogens

orcid.org/0000-0003-2946-9497

Michael Malim

Editor-in-Chief

PLOS Pathogens

orcid.org/0000-0002-7699-2064

***********************************************************

Reviewer Comments (if any, and for reference):

# Editor report: Acceptance letter

**DOI:** 10.1371/journal.ppat.1012938.r005

**Authors:** Margaret A Phillips, Dawn M Wetzel

Dear Mrs Boité,

We are delighted to inform you that your manuscript, "Gene deletion as a possible strategy adopted by New World Leishmania infantum to maximize geographic dispersion," has been formally accepted for publication in PLOS Pathogens.

We have now passed your article onto the PLOS Production Department who will complete the rest of the pre-publication process. All authors will receive a confirmation email upon publication.

The corresponding author will soon be receiving a typeset proof for review, to ensure errors have not been introduced during production. Please review the PDF proof of your manuscript carefully, as this is the last chance to correct any scientific or type-setting errors. Please note that major changes, or those which affect the scientific understanding of the work, will likely cause delays to the publication date of your manuscript. Note: Proofs for Front Matter articles (Pearls, Reviews, Opinions, etc...) are generated on a different schedule and may not be made available as quickly.

Soon after your final files are uploaded, the early version of your manuscript, if you opted to have an early version of your article, will be published online. The date of the early version will be your article's publication date. The final article will be published to the same URL, and all versions of the paper will be accessible to readers.

Thank you again for supporting open-access publishing; we are looking forward to publishing your work in PLOS Pathogens.

Best regards,

Sumita Bhaduri-McIntosh

Editor-in-Chief

PLOS Pathogens

orcid.org/0000-0003-2946-9497

Michael Malim

Editor-in-Chief

PLOS Pathogens

orcid.org/0000-0002-7699-2064
